# The pH-Responsive PacC Transcription Factor of *Aspergillus fumigatus* Governs Epithelial Entry and Tissue Invasion during Pulmonary Aspergillosis

**DOI:** 10.1371/journal.ppat.1004413

**Published:** 2014-10-16

**Authors:** Margherita Bertuzzi, Markus Schrettl, Laura Alcazar-Fuoli, Timothy C. Cairns, Alberto Muñoz, Louise A. Walker, Susanne Herbst, Maryam Safari, Angela M. Cheverton, Dan Chen, Hong Liu, Shinobu Saijo, Natalie D. Fedorova, Darius Armstrong-James, Carol A. Munro, Nick D. Read, Scott G. Filler, Eduardo A. Espeso, William C. Nierman, Hubertus Haas, Elaine M. Bignell

**Affiliations:** 1 Institute for Inflammation and Repair, University of Manchester, Manchester, United Kingdom; 2 Division of Molecular Biology/Biocenter, Medical University Innsbruck, Innsbruck, Austria; 3 Sandoz GmbH, Kundl, Austria; 4 National Centre for Microbiology, Instituto de Salud Carlos III, Madrid, Spain; 5 College of Life and Environmental Sciences, University of Exeter, Exeter, United Kingdom; 6 School of Medical Sciences, University of Aberdeen, Aberdeen, United Kingdom; 7 Centre for Molecular Bacteriology & Infection, Imperial College London, London, United Kingdom; 8 Lab of Viral Diseases, National Institute of Allergy and Infectious Diseases, National Institute of Health, Bethesda, Maryland, United States of America; 9 David Geffen School of Medicine at University of California, Los Angeles, Los Angeles Biomedical Research Institute at Harbor, University of California, Los Angeles Medical Center, Los Angeles, California, United States of America; 10 Medical Mycology Research Center, Chiba University, Chiba, Japan; 11 Department of Molecular and Cellular Biology, Centro de Investigaciones Biológicas (C.S.I.C.), Madrid, Spain; University of Michigan, United States of America

## Abstract

Destruction of the pulmonary epithelium is a major feature of lung diseases caused by the mould pathogen *Aspergillus fumigatus*. Although it is widely postulated that tissue invasion is governed by fungal proteases, *A. fumigatus* mutants lacking individual or multiple enzymes remain fully invasive, suggesting a concomitant requirement for other pathogenic activities during host invasion. In this study we discovered, and exploited, a novel, tissue non-invasive, phenotype in *A. fumigatus* mutants lacking the pH-responsive transcription factor PacC. Our study revealed a novel mode of epithelial entry, occurring in a cell wall-dependent manner prior to protease production, and via the Dectin-1 β-glucan receptor. *ΔpacC* mutants are defective in both contact-mediated epithelial entry and protease expression, and significantly attenuated for pathogenicity in leukopenic mice. We combined murine infection modelling, *in vivo* transcriptomics, and *in vitro* infections of human alveolar epithelia, to delineate two major, and sequentially acting, PacC-dependent processes impacting epithelial integrity *in vitro* and tissue invasion in the whole animal. We demonstrate that *A. fumigatus* spores and germlings are internalised by epithelial cells in a contact-, actin-, cell wall- and Dectin-1 dependent manner and *ΔpacC* mutants, which aberrantly remodel the cell wall during germinative growth, are unable to gain entry into epithelial cells, both *in vitro* and *in vivo*. We further show that PacC acts as a global transcriptional regulator of secreted molecules during growth in the leukopenic mammalian lung, and profile the full cohort of secreted gene products expressed during invasive infection. Our study reveals a combinatorial mode of tissue entry dependent upon sequential, and mechanistically distinct, perturbations of the pulmonary epithelium and demonstrates, for the first time a protective role for Dectin-1 blockade in epithelial defences. Infecting *ΔpacC* mutants are hypersensitive to cell wall-active antifungal agents highlighting the value of PacC signalling as a target for antifungal therapy.

## Author Summary

Inhaled spores of the pathogenic mould *Aspergillus fumigatus* cause fungal lung infections in humans having immune defects. *A. fumigatus* spores germinate within the immunocompromised lung, producing invasively growing, elongated cells called hyphae. Hyphae degrade the surrounding pulmonary tissue, a process thought to be caused by secreted fungal enzymes; however, *A. fumigatus* mutants lacking one or more protease activities retain fully invasive phenotypes in mouse models of disease. Here we report the first discovery of a non-invasive *A. fumigatus* mutant, which lacks a pH-responsive transcription factor PacC. Using global transcriptional profiling of wild type and mutant isolates, and *in vitro* pulmonary invasion assays, we established that loss of PacC leads to a compound non-invasive phenotype characterised by deficits in both contact-mediated epithelial entry and protease expression. Consistent with an important role for epithelial entry in promoting invasive disease in mammalian tissues, PacC mutants remain surface-localised on mammalian epithelia, both *in vitro* and *in vivo*. Our study sets a new precedent for involvement of both host and pathogen activities in promoting epithelial invasion by *A. fumigatus* and supports a model wherein fungal protease activity acting subsequently to, or in parallel with, host-mediated epithelial entry provides the mechanistic basis for tissue invasion.

## Introduction

Spores of the mould pathogen *Aspergillus fumigatus* are agents of multiple human diseases, most of which initiate with inhalation of fungal spores and, dependent upon host immune status, compromise pulmonary integrity. Amongst the resultant diseases, invasive aspergillosis (IA) exerts the highest fatal toll, resulting globally in an estimated 200,000 deaths per annum [1]. Recipients of allogenic hematopoietic stem cell- or solid organ transplants, are particularly susceptible to IA which accounts for 43% and 19% of all invasive fungal infections in these cohorts and causes 58% and 34% mortality, respectively, at 12 weeks post-transplant [2]–[4]. Structural or immunological lung defects also lead to chronic, semi-invasive, pulmonary aspergillosis (CPA) having a 5 year mortality of 50% [5]. Amongst more than 200 Aspergillus species, *Aspergillus fumigatus* accounts for the majority of these diseases [6].

In diseases caused by *A. fumigatus* the initiating host-pathogen interaction occurs at pulmonary epithelia where inhaled spores can exit from dormancy, swell and generate invasive cells called hyphae, which traverse the lung epithelium. A key pathological feature of invasive- and semi-invasive aspergilloses is the destruction of the lung parenchyma, hypothesised to be governed by proteolytic enzymes secreted by the invading pathogen. Exposure of *in vitro*-cultured bronchial, or alveolar, epithelial cells (ECs) to fungal culture supernatants has revealed a role for fungal proteases in destruction of the mammalian F-actin cytoskeleton and loss of focal adhesion [7]–[9]. However, in whole animal studies of disease, it has not been possible to attribute lung damage solely to the activity of fungal proteases since *A. fumigatus* mutants lacking individual, or multiple, enzymes retain the ability to cause fatal invasive infections in immunocompromised hosts [9]–[15].

The interaction of *A. fumigatus* spores with alveolar epithelia can result in the internalisation of spores [16]–[18] but the role of this process in disease outcome remains unknown. Cells of the A549 pneumocyte cell line [18] and 16HBE14o- transformed human bronchial epithelial cells [19] internalise 30–50% of encountered spores, via an actin-dependent mechanism. Whilst the vast majority of internalised spores are killed, a small proportion (∼3%) survives and germinates inside acidic organelles [20]. This has prompted hypotheses of latent occupation of host epithelia by *A. fumigatus* spores, which might thereby evade host immunity and initiate disseminated infections (as reviewed by Osherov, 2011 [21]). Conversely, a curative role for epithelial activities is strongly supported by the observations of Chaudhary *et al.*, 2012 who reported that bronchial ECs harbouring a CFTR mutation (ΔF508) demonstrate impaired uptake and killing of conidia [22]. The impact of EC-mediated activities upon disease outcomes in whole animal models of infection is presently unclear.

In fungi a conserved regulatory pathway governs the pH-dependent expression of secreted proteins and adaptation to alkaline stress [23]–[27]. Acting via PacC/Rim101 transcription factors, this environmental adaptation mechanism promotes the energy-efficient production of exported enzymes and metabolites, and has a demonstrated role in the pathogenicity of *Candida albicans* [25], [28] and *Aspergillus nidulans* [29]. Analysis of the transcriptomic response of invasive *A. fumigatus* hyphae to the mammalian pulmonary niche, identified 102 alkaline-responsive gene functions as being upregulated in leukopenic mice [30]. We therefore hypothesised that in *A. fumigatus*, PacC would be important for colonisation of the mildly alkaline murine lung. In this study we describe a functional genomics analysis of PacC-mediated activities which govern pathogenicity in mice. Unexpectedly, we discovered that PacC null mutants exhibit an unprecedented non-invasive phenotype, which is not an artefact of defective fungal growth, and can be recapitulated *in vitro* using cultured epithelia. The capacity to invade host tissues is therefore a genetically regulated trait, requiring PacC regulatory control.

In this study we exploited the differentially invasive properties of wild-type and *ΔpacC* isolates, to address the cellular and molecular basis of pathogen-mediated epithelial damage during *Aspergillus* infections. This revealed a novel mode of epithelial entry, occurring in a cell wall-dependent manner prior to protease production, and via the Dectin-1 β-glucan receptor. Our findings reveal novel mechanistic insights, having direct relevance to infection of whole animals, which will focus the onward study of *A. fumigatus*-mediated lung diseases upon dissecting the synergistic and/or additive impacts of temporally distinct aspects of the host-pathogen interaction at pulmonary epithelia. The multiple deficits in pathogenic activities and heightened sensitivity to echinocandin drugs, observed in *ΔpacC* isolates, highlight the potential of this receptor-mediated fungal signalling mechanism as a target for antifungal therapies.

## Results

### The pH-responsive *A. fumigatus* transcription factor PacC is required for epithelial invasion and pathogenicity in leukopenic mice

**Figure 1:**
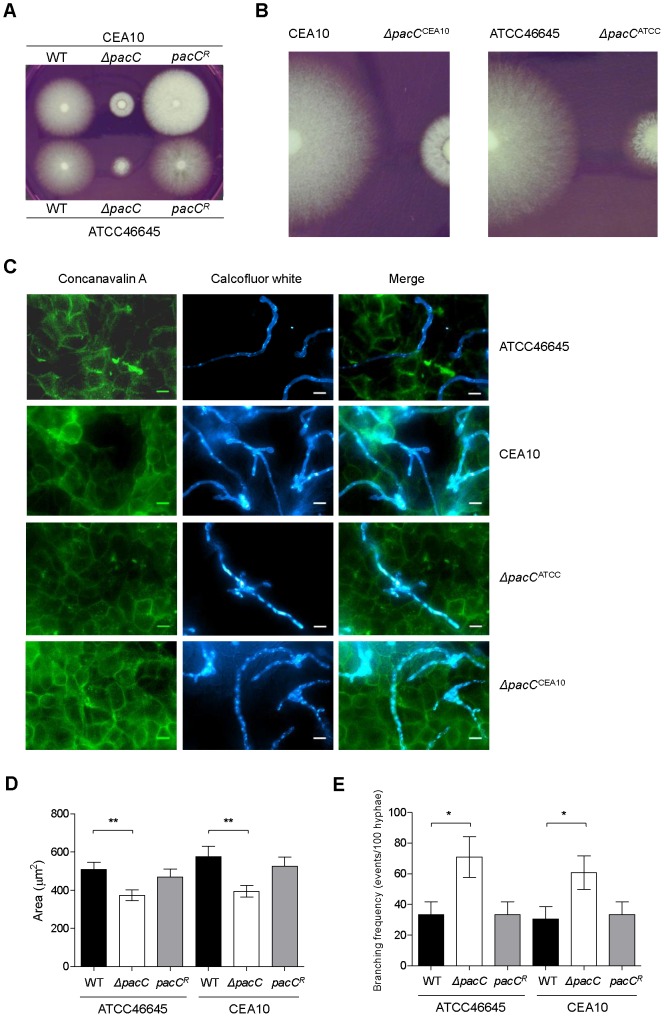
Impact of *pacC* deletion upon *A. fumigatus* growth. **(A and B)** Colonial growth phenotypes on supplemented DMEM, 48 hr of growth, 10^3^ conidia. **(C)** Immunofluorescence-mediated visualisation of *A. fumigatus* growth in co-culture with A549 epithelial cells, 16 hours, supplemented DMEM pH 7.4. Fungal cells are labelled with calcoflour white and host cells are labelled with FITC-conjugated concanavalin A, 10^5^ spores/ml, 200× magnification. **(D)** Quantitation of cell size (μM^2^) at 16 hr of co-culture with A549 cells, 10^4^ spores/ml, growth conditions as for panel C. **(E)** Branching frequency of *A. fumigatus* hyphae, 10^4^ spores/ml, growth conditions as for panel C.

To characterise the role of the *A. fumigatus* PacC transcription factor (AFUA_3G11970) in pathogenicity we constructed null and complemented alleles in two distinct *A. fumigatus* clinical isolates CEA10 and ATCC46645 (Figure S1). Relative to non-mutated isolates, PacC null mutants assumed a compact colonial phenotype on supplemented solid DMEM medium pH 7.4 (Figure 1A) which, in contrast to colonies of wild type isolates, lacked peripheral invasive hyphae, and were composed of a denser hyphal network indicative of a hyperbranching morphology (Figure 1B). This compact morphology was pH-independent, being also observed in colonies grown on Aspergillus complete medium pH 6.5 (Figure S2A), where sporulation and pigmentation of *ΔpacC* mutants was equivalent to that of the wild type. To assess pH sensitivity of the *ΔpacC* mutants we examined, on pH-buffered minimal media, the extent of radial growth at pHs 8.0 and 7.2, relative to growth at pH 6.5 (Figures S2B and S2C respectively). Consistent with a role for PacC in alkaline adaptation, *ΔpacC* isolates suffered growth impairment at pH 8.0, achieving approximately 10–20% of the radial growth attained at pH 6.5 (Figure S2B), compared with 40% achieved by wild-type and reconstituted strains. However, sensitivity of *ΔpacC* isolates to growth at pH 7.2, which approximates the pH of the mammalian lung, did not differ from that of the wild type isolates (Figure S2C).

To orchestrate alkaline adaptation, cytoplasmically localised PacC/Rim101 transcription factors must undergo pH-dependent proteolytic cleavage and nuclear entry [31]. Concordant with this model of transcription factor activation, we detected, by electrophoretic mobility shift assay (EMSA), several PacC retardation complexes, the relative quantities of which were altered under acidic and alkaline growth conditions (Figures S3A and S3B). Relative to growth at acidic pH, processed, activated forms of PacC increased in abundance upon shifting to alkaline conditions (Figures S3A and S3B). These findings are consistent with a pH-responsive mode of PacC activation, and with a conserved role for the *A. fumigatus* transcription factor in alkaline adaptation.

The mammalian pulmonary niche exerts multiple physiological stresses upon invading pathogens, including mildly alkaline pH, hypoxia, and iron-, zinc- and nutrient limitation [30], [32], [33]. To compare the growth rates of wild type and *ΔpacC* hyphae in a physiologically relevant setting, we devised an *in vitro* epithelial infection assay (Figure 1C), comprising monolayers of A549 alveolar epithelial cells cultured in supplemented DMEM medium (pH 7.4). We used this assay to assess the growth rates of wild type and *ΔpacC* isolates, under 5% CO_2_ (Figure 1C). To promote the visualisation of, and distinction between, fungal and host cells we stained host cells with FITC-labelled concavalin A and fungal cells with calcofluor white. We then performed a quantitative analysis of fungal growth rate (Figure 1D) and hyphal branching (Figure 1E) via immunofluorescence microscopy. Inspection of the infected monolayers revealed significant hyphal growth of both wild type and *ΔpacC* isolates (Figure 1C), but quantitation of hyphal branching frequency revealed an approximately doubled frequency of hyphal branching amongst *ΔpacC* isolates relative to wild type. To normalise for heightened branching during quantitation of hyphal growth rates we performed a comparative assessment of individual cell sizes by enumerating the number of pixels per fungal particle. This analysis, revealed that the cross-sectional area of wild type and *ΔpacC* cells approximates 540 versus 387 µm^2^ respectively (Figure 1D) which, assuming a uniform hyphal radius for wild type and mutant cells of approximately 3 µm, equates to a maximum deviance of 20 µm in length after 16 hours of *in vitro* co-culture with mammalian epithelia. On this mathematical basis, the median length of an unbranched *ΔpacC* hypha would be ∼ 30 µm. Given that ∼ 50% of *ΔpacC* hyphae remain unbranched, and the thickness of the alveolar epithelium is comparatively tiny, the observed differences in hyphal length between wild type and mutant hyphae are insufficient to explain the non-invasive phenotype of the *ΔpacC* mutants.

**Figure 2:**
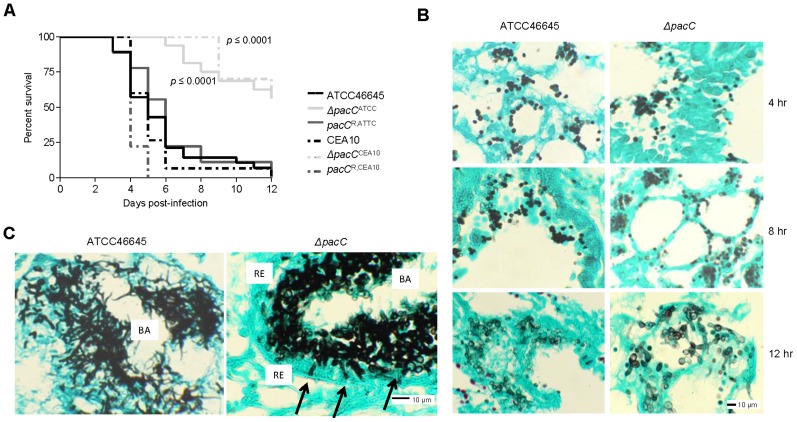
PacC is required for pathogenicity and epithelial invasion in leukopenic mice. **(A)** Kaplan-Meier curve for murine survival (n ≥ 9) after infection with 6 × 10^5^ and 5 × 10^4^ spores for ATCC46645 and CEA10 respectively. **(B)** Histopathology of leukopenic murine lungs after 4, 8, 12, hr of infection with ATCC46645 and *ΔpacC* ^ATCC^ (10^8^ spores), Grocott's Methenamine silver (GMS) and light green staining, 200× magnification. **(C)** *ΔpacC* ^ATCC^ hyphae (black arrows) are unable to penetrate the respiratory epithelium (RE), BA indicates bronchial airway space, 20 hr post-infection, 400× magnification.

Leukopenia is an important risk factor for IA in humans, and cyclophosphamide-induced leukocyte depletion renders mice highly susceptible to pulmonary infection [34]. To assess the role of *A. fumigatus* PacC in pathogenicity we assessed the survival of leukopenic mice following infection, via the intranasal route, with spores of wild-type, *ΔpacC* or reconstituted isolates (Figure 2A). Relative to wild-type strains, *ΔpacC* ^ATCC^ and *ΔpacC* ^CEA10^ mutants were significantly attenuated for virulence (Figure 2A). At day 6 post-infection 100% of mice infected with the *ΔpacC* mutants remained alive while 78% and 92% of mice infected with wild type (ATCC46645 and CEA10 respectively) isolates were dead.

Histological analysis of infected lung tissues revealed significant differences between mutant and wild-type isolates by 20 hours post-infection (Figures 2B and C). While *ΔpacC* spores appeared to swell and form primary germ tubes by 12 hours post-infection (Figure 2B), penetration of the lung epithelium was not evident and, despite being competent in hyphal production in the murine airway, *ΔpacC* germlings remain contained within the epithelial boundary of the airspace at 20 hours post-infection (Figure 2C). Thus, *ΔpacC* germlings in leukopenic hosts exhibited a marked tissue non-invasive phenotype (Figures 2B and 2C).

**Figure 3:**
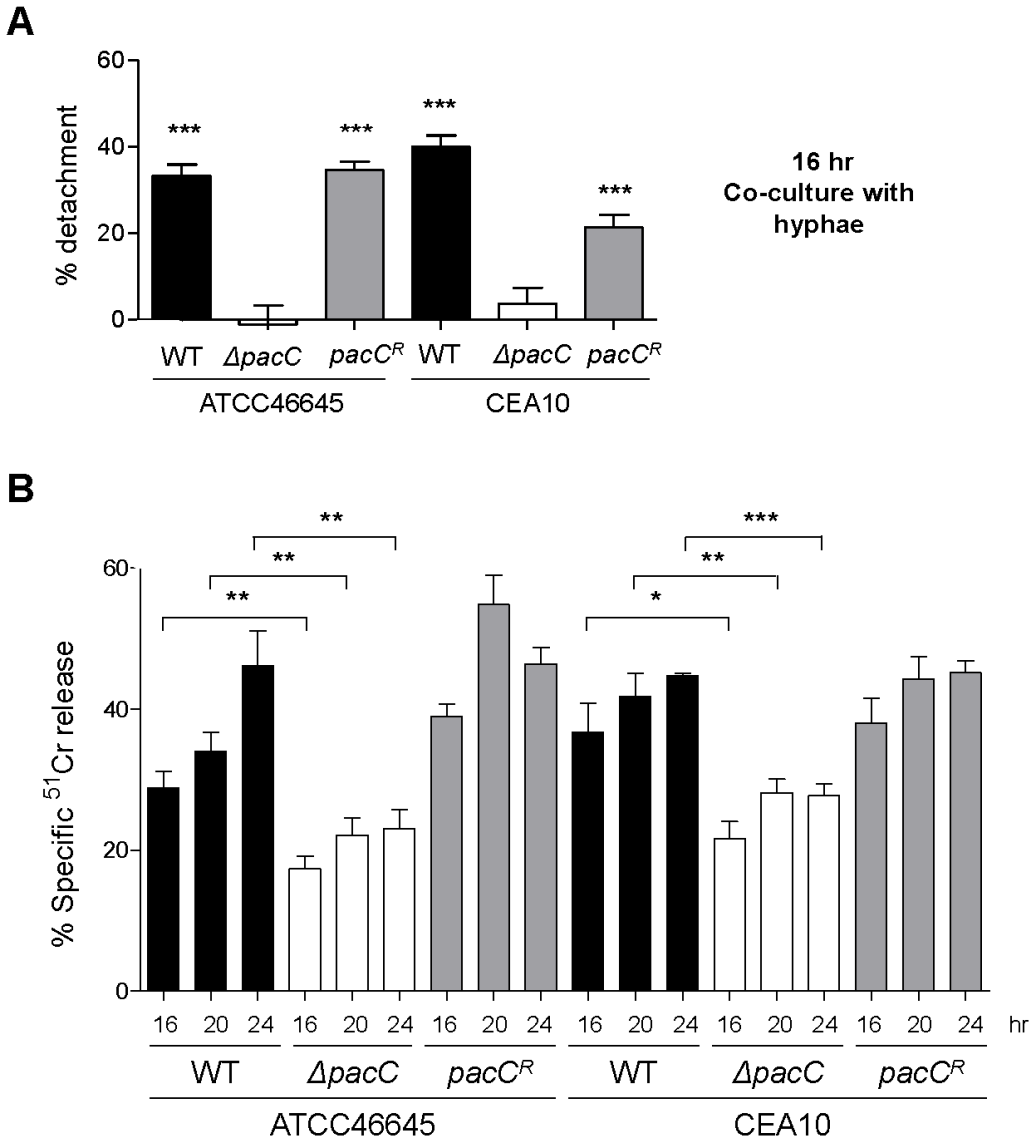
Quantitative analysis of epithelial integrity. **(A)** Percent detachment, relative to PBS challenge, quantified from a similar experiment as that depicted in Figure 1C. Significance was calculated relative to PBS-challenged A549 cells. **(B)** Quantification of A549 damage by ^51^Cr release assay upon infection with *A. fumigatus* (5 × 10^5^ spores/ml) for 16, 20 and 24 hr. A and B: technical and biological triplicate, unpaired *t* test. *** *p*<0.001, ** 0.001 <*p*<0.01, and * 0.01 <*p*<0.05.

To further characterise the non-invasive phenotype of *ΔpacC* isolates we used our *in vitro* co-culture assay (Figure 1C) to examine integrity of A549 alveolar epithelial monolayers following 16 hours co-culture with wild-type or *ΔpacC* isolates by enumeration of detaching epithelial cells. In monolayers infected with wild-type strains, extensive rounding and detachment of up to 40% of host cells was demonstrable, resulting in observable destruction of the epithelial monolayer (Figures 1C and 3A). However, similar to PBS-challenged monolayers, infection with *ΔpacC* mutants led to detachment of less than 5% of monolayer cells (Figures 1C and 3A). Although *ΔpacC* hyphae achieved similar hyphal growth rates (Figure 1D) and epithelial coverage (Figure 1C) to isogenic wild type isolates, *ΔpacC* hyphae were found to navigate the surface of the epithelial monolayer without effecting cellular detachment (Figure 1C).

To further characterise this deficit in epithelial damage, a modified ^51^Cr release assay [35], adopting a similar time course of infection, was utilised to quantitatively assess epithelial cell lysis upon infection (Figure 3B). Concordant with cell detachment assays, *ΔpacC* mutants reproducibly failed to fully elicit epithelial damage. Taken together these findings demonstrate that *ΔpacC* hyphae fail to elicit disintegration of alveolar epithelia during *in vitro* culture at pH 7.4 despite achieving similar growth rates as wild-type isolates (Figures 1C and D and 3A and B).

Several observations argue strongly against a trivial pH-dependent growth defect as the basis for the *ΔpacC* virulence defect. First, in *A. fumigatus*, a compact colonial morphology is not a robust correlate of reduced virulence. For example, despite compact colonial morphology and a highly branching mode of growth, null mutants lacking the ChsG chitin synthase remain fully virulent in an inhalational model of infection [36]. Second, in *A. fumigatus*, *in vitro* alkaline sensitivity is not a robust correlate of reduced virulence. For example, mutants lacking the mitogen-activated kinase MpkA, demonstrate more severe radial growth defects than *ΔpacC* mutants in Aspergillus minimal medium [37] and exhibit highly alkaline sensitive phenotypes [38], but retain full virulence in a low dose inhalational model of aspergillosis. Third, during *in vitro* cell culture with A549 cells (pH 7.4), hyphae of *ΔpacC* mutants achieve similar growth rates to that of isogenic wild type isolates (Figures 1C and D). Fourth, despite achieving similar growth rates to wild type hyphae during *in vitro* cell culture with A549 cells (pH 7.4), hyphae of *ΔpacC* mutants fail to elicit epithelial decay (Figures 1C, 3A and B). Finally, hyphae of *ΔpacC* mutants fail to traverse the murine epithelium and are strictly confined to the airway (Figure 2C). *ΔpacC* mutants are the first-reported non-invasive *A. fumigatus* mutants, revealing that tissue invasion is a genetically regulated trait under PacC regulatory control. We therefore exploited the differentially invasive properties of wild type and *ΔpacC* isolates to seek a more detailed mechanistic understanding of tissue invasive growth in this pathogen.

### PacC modulates expression of genes encoding secreted enzymes and cell wall- and gliotoxin biosynthetic activities during invasive aspergillosis

Previously, we devised a strategy for analysing *A. fumigatus* gene expression during initiation of murine aspergillosis [30]. Here we applied a similar approach, this time performing time-series analyses (4, 8, 12 and 16 hours) of *A. fumigatus* gene expression. This permitted the capture of stage-specific gene expression during invasive colonisation of the leukopenic murine lung, the first reported longitudinal study of gene expression during mammalian pulmonary infection. We adopted a comprehensive experimental design (Figure S4A), incorporating 12 competitive hybridisations and 12 flip-dye experiments. This permitted the analysis of stage-specific gene expression in both infecting wild-type ATCC46645 (Figure S4B) and *ΔpacC* ^ATCC^ isolates (Figure S4C), as well as the directly comparative analysis of gene expression, by time-point, for the wild type and *ΔpacC* ^ATCC^ mutants (Figure S4D).

Relative to ungerminated spores, transcript profiling of wild-type gene expression revealed a total of 3733 genes, (log_2_≥ +/−1.5), which were differentially expressed at a minimum of one time-point during invasive infection. The differentially regulated genes were assigned to three cohorts, corresponding to (i) genes consistently up- or down-regulated across the time series; or differentially regulated during (ii) early (4, 8 and 12 hours) or (iii) late (12 and 16 hours) phases (Dataset S1). This revealed respiration, metabolism and amino acid biosynthesis as being prioritised during early infection of the leukopenic host, while cation transport, secondary metabolism and iron metabolism were subsequently emphasised during commencement of invasive growth (Dataset S1). Throughout the time series of growth in the host, upregulated expression of secreted gene products remained highly significant. A comprehensive functional, and statistical, analysis of differentially regulated gene products is provided in Dataset S1.

Directly comparative analysis of *ΔpacC* ^ATCC^ and ATCC46645 activities (Dataset S2) revealed 1116 genes to be differentially expressed. Of these, 577 were up-regulated and 539 were down-regulated in the *ΔpacC* ^ATCC^ isolate, relative to the wild type isolate, in at least one time point of the analysis. Scrutiny of the datasets revealed dysregulated expression of secreted protein gene products, defined as having predicted signal peptide motifs (Figure S5), cell wall biosynthetic enzymes (Figure S6), and gliotoxin biosynthetic genes (Figure S7) during infections caused by the *ΔpacC* ^ATCC^ isolate. A comprehensive functional, and statistical, analysis of differentially regulated gene products is provided in Dataset S2. The differential regulation of 5 genes was independently validated by quantitative PCR (Figure S8).

### *A. fumigatus* perturbs epithelial integrity via distinct, and sequentially implemented, mechanisms which are defective in *ΔpacC* isolates

**Figure 4:**
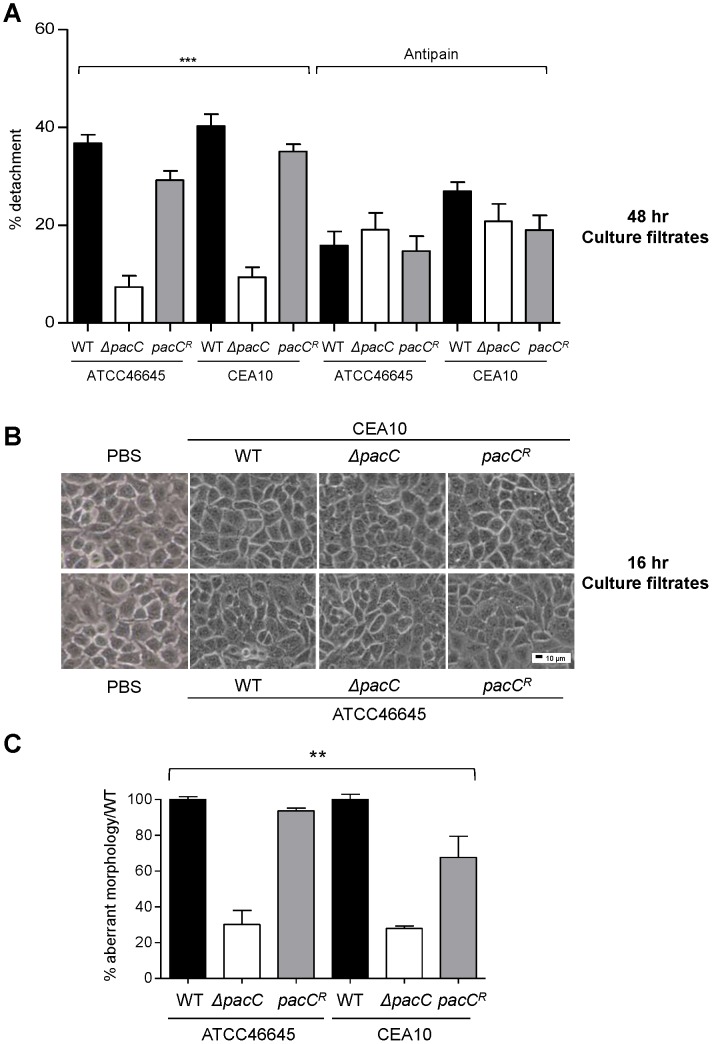
Distinct and sequential PacC-dependent activities elicit epithelial decay. **(A)** Percent detachment, relative to PBS challenge, of A549 cells exposed to antipain-treated and non-treated fungal culture supernatants (48 hr cultures, 10^5^ spores/ml). **(B)** Integrity of A549 monolayers following exposure to fungal culture supernatants (16 hr cultures, 10^5^ spores/ml). 200× magnification. **(C)** Percent aberrant morphology, amongst fungus-proximal ECs, per unit of hyphal length, relative to wild-type challenged monolayers. Monolayers were incubated for 18 hr or 36 hr (*ΔpacC* mutants). A and B: technical and biological triplicates and C: performed twice in triplicate, 1-way ANOVA test, *** *p*<0.001 and ** 0.001 <*p*<0.01.

*A. fumigatus* spores adhere rapidly (within 30 minutes) to lung pneumocytes and become quickly internalised and killed [16]–[18], [20]. In response to challenge with *A. fumigatus* conidia, host injury can be observed as cell rounding and detachment from monolayers [16]–[18], [20], and cytoskeletal fibres of lung pneumocytes suffer major reorganisation, an effect which can be blocked by antipain-mediated protease inhibition [8]. We found secreted factors to be the major functional cohort amongst those aberrantly regulated during *ΔpacC* infections (Figure S5, and Dataset S2). To assess the role of secreted factors in epithelial disintegration we exposed A549 monolayers to *A. fumigatus* culture filtrates derived from young (16 hours), or mature (48 hours), wild-type or *ΔpacC* cultures grown in supplemented DMEM culture medium. Filtrates obtained from mature cultures of wild-type or reconstituted *A. fumigatus* isolates, prompted significant reductions (∼30–40%) in the numbers of adherent cells after 20 hours of co-incubation with alveolar epithelia (Figure 4A). In contrast, filtrates derived from mature *ΔpacC* cultures led to detachment of less than 10% of monolayer cells (Figure 4A). Concordant with a protease-mediated basis for epithelial destruction, pre-treatment of wild-type *A. fumigatus* culture filtrates with the protease inhibitor antipain [8] reduced cellular detachment by up to 50% (Figure 4A). To further probe protease production by wild type and mutant isolates, we used a qualitative assay based upon the clearance of gelatin from the surface of unprocessed X-ray film [39]. In agreement with our assays of epithelial degradation (Figure 4A) we detected gelatin-degrading activity in filtrates of mature wild type and reconstituted *A. fumigatus* cultures, which was absent in cultures from *ΔpacC* isolates (Figure S9).

Together, these findings are consistent with the release of a damaging proteolytic entity by mature *A. fumigatus* hyphae, the production of which is dependent upon PacC-mediated signalling. Notably, epithelia challenged with *ΔpacC* culture supernatants were somewhat protected by pretreatment of fungal extracts with antipain (Figure 4A). This might indicate the production of a protective, host-derived enzyme which is degraded or inactivated by culture filtrates of wild type *A. fumigatus* isolates, or the existence of a protective, host-derived enzyme whose action, in this assay, is masked by the high degree of epithelial detachment imposed by wild type isolates.

Critically, our analysis of filtrates obtained from younger fungal cultures (16 hours) revealed a novel finding. Regardless of the fungal strain tested, exposure to filtrates from young cultures did not impact monolayer integrity (Figure 4B). This finding suggested that epithelial disintegration occurring at 16 hours of spore and EC co-incubation (Figure 4B) requires direct interaction between host and pathogen cells and represents a genetically regulated fungal assault upon epithelial integrity which is temporally, and mechanistically distinct from protease-mediated damage. Further, that this damaging interaction between host and pathogen cells occurs during immediate proximity between host and pathogen cells, most likely in a contact-dependent manner. To substantiate this view we reiterated the detachment analysis, this time omitting monolayer washing to analyse only host cells directly contacting the pathogen (Figure 4C). A549 cells in contact with *ΔpacC* hyphae underwent significantly less rounding and detachment than those contacting hyphae of wild-type or reconstituted isolates (Figure 4C). Taken together, these data reveal that *A. fumigatus* elicits host damage in a biphasic manner and, that the pH-responsive transcription factor PacC governs functions required for epithelial disintegration during both early- and late-phases of the host-pathogen interaction.

### *A. fumigatus* causes epithelial disintegration in a cell wall-dependent manner

**Figure 5:**
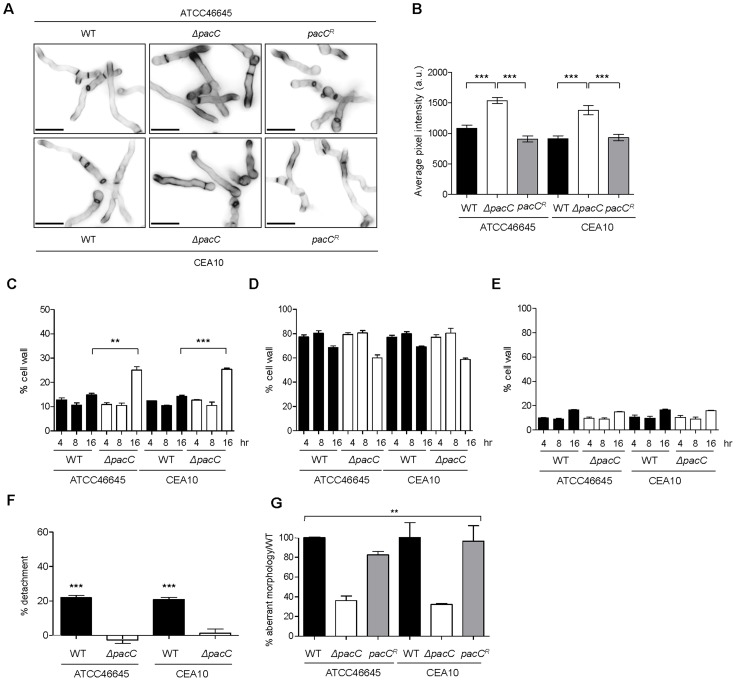
Aberrant morphology of *A. fumigatus ΔpacC* mutants impacts contact-mediated epithelial decay. **(A)** Calcofluor white staining of *A. fumigatus* strains after growth for 17–18 hr in AMM (pH 6.5) at 28°C. **(B)** Quantification of calcofluor white-mediated fluorescence (arbitrary units) as measured from panel A. **(C)** Percentage, in cell wall extracts relative to total weight, of chitin. **(D)** Percentage, in cell wall extracts relative to total weight, of glucan. **(E)** Percentage, in cell wall extracts relative to total weight, of mannan. **(F)** Percent detachment, relative to PBS challenge, of A549 cells after co-incubation with cell wall extracts (10 µg/ml). Significance was calculated relative to PBS-challenged monolayers. **(G)** Percent aberrant morphology, amongst thimerosal-killed fungus-proximal ECs, per unit of hyphal length, relative to wild-type challenged monolayers. B and C: biological and technical triplicates. D and E: performed twice in triplicate. B, C and D: unpaired *t* test. E: 1-way ANOVA test. *** *p*<0.001 and ** 0.001<*p*<0.01.

Amongst the functional cohorts aberrantly regulated during *ΔpacC* ^ATCC^ infections (Figures S5–S7 and Dataset S2) cell wall biosynthesis offered a plausible mechanism for contact-dependent host damage. To analyse cell wall compositions of mutant and wild-type isolates, strains were stained with the chitin-binding agent calcofluor white (CFW). Microscopic examination revealed intensified CFW-staining of *ΔpacC* germ tube tips relative to those of the parental isolates (Figure 5A) and quantitative analysis of fluorescence intensities revealed significantly higher CFW in *ΔpacC* germ tubes (Figure 5B). In addition, electron microscopy showed a thickened cell wall in the *ΔpacC* ^ATCC^ mutant, which was highly evident after 16 hours of growth (Figure S10). Hyphal cell wall composition (Figures 5C, D and E) was assessed by high-performance anion exchange chromatography with pulsed amperometric detection (HPAEC-PAD). After 16 hours of growth, cell wall chitin content was found to be 20% higher in extracts from *ΔpacC* isolates, relative to wild-type cells (Figure 5C). The quantity of cell wall glucan and mannan was measured as equivalent between mutant and wild type cells (Figures 5D and 5E).

To probe the contribution of cell wall components to epithelial cell detachment, we exposed A549 monolayers to cell wall extracts. This revealed that, relative to the vehicle control, the percentage of adherent cells was drastically reduced upon exposure to cell wall extracts from wild-type isolates, an effect which was not elicited by *ΔpacC* cell wall extracts (Figure 5F).

If detachment of alveolar epithelial cells occurs via a cell wall-mediated mechanism, infection with dead hyphae would be predicted also to cause cellular detachment. To test this, A549 monolayers were incubated with thimerosal-killed hyphae and cell detachment, per unit of hyphal length, was measured from unwashed monolayers. Killed hyphae from parental isolates caused damage to the epithelial monolayer independently of fungal viability, an effect which was significantly impaired in monolayers incubated with killed *ΔpacC* hyphae (Figure 5G). In conclusion, epithelial detachment elicited early in the interaction between *A. fumigatus* and host cells requires contact between host and pathogen and occurs independently of fungal viability. Crucially, the inability of killed *ΔpacC* hyphae to injure epithelial monolayers eliminated the trivial possibility that mere collision between host and fungal cells can account for loss of monolayer integrity.

### Dectin-1, in alveolar epithelial cells, promotes internalisation of *A. fumigatus* spores

The mammalian C-type lectin receptor Dectin-1, is predominantly expressed by myeloid cells and recognizes a variety of fungal β-1,3-linked and β-1,6-linked glucans [40]–[42]. Recent transcriptional and immunohistochemical analyses have revealed Dectin-1 gene expression in bronchiolar epithelia, and alveolar type II cells (ATIIs) of murine lungs [43] and Han *et al.*, showed that A549 cells internalise germinated *A. fumigatus* spores in a phospholipase D-dependent manner, a process inhibited by an anti-Dectin-1 antibody [44]. Given these observations and aberrant cell wall remodelling in (Figure 5), and epithelial damage by (Figure 4), *ΔpacC* mutants we hypothesised non- or reduced involvement of Dectin-1 in promoting *A. fumigatus ΔpacC* spore recognition and internalisation.

**Figure 6:**
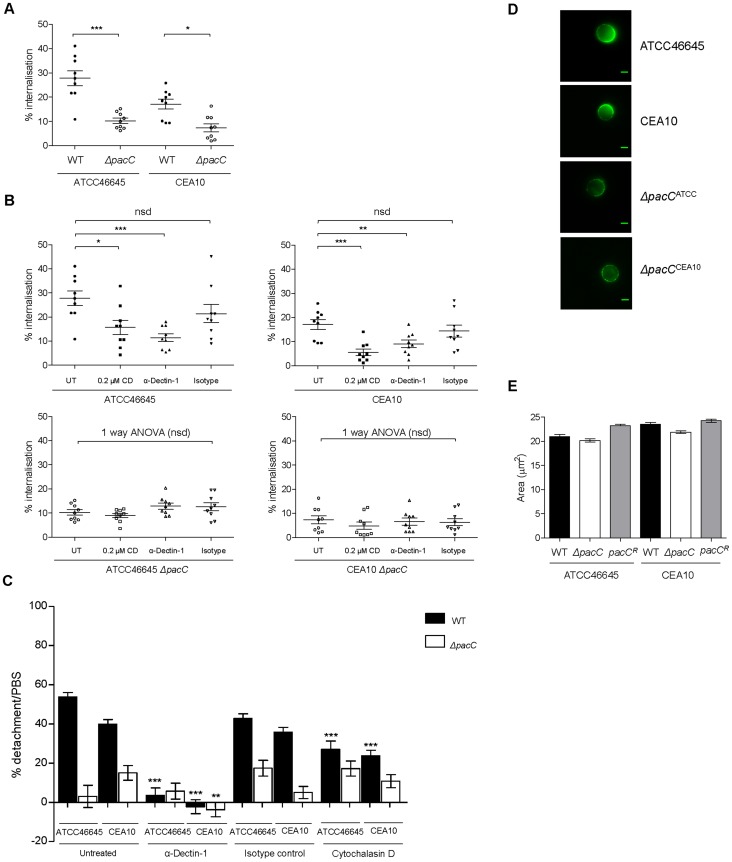
*ΔpacC* mutants are less avidly internalised by A549 and spore internalisation depends on Dectin-1. **(A)** Percent internalisation by nystatin protection assay. Epithelial monolayers were incubated with 10^6^ spores/ml for 4 hr, n  =  9 for wild types and *ΔpacC*. **(B)** Percent internalisation, when blocking the Dectin-1 receptor (0.3 µg/ml α-Dectin-1 antibody) or actin-mediated internalisation (0.2 µM CD), or in the presence of an isotype control antibody (0.3 µg/ml) technical and biological duplicates. **(C)** Percent detachment, relative to PBS challenge, of A549 upon infection with *A. fumigatus* (10^5^ spores/ml) for 16 hr and inhibition of actin-mediated internalisation via Cytochalasin D (CD, 0.2 µM), when blocking the Dectin-1 receptor (0.3 µg/ml α-Dectin-1 antibody) or in the presence of an isotype control antibody (0.3 µg/ml), technical and biological triplicates. **(D)** Use of a soluble Fc Dectin-1 protein for immunofluorescence-mediated imaging of β-glucan in *A. fumigatus* spores, 4 hr, supplemented DMEM. **(E)** *A. fumigatus* spore size (μM^2^), 4 hr, supplemented DMEM. A, B, C: unpaired *t* test (unless otherwise indicated) *** *p*<0.001, ** 0.001<*p*<0.01, and * 0.01<*p*<0.05.

Epithelial cells have been shown to internalise and kill up to 50% of the *A. fumigatus* spores they come into contact with [16]–[19], [22]. We therefore hypothesised that contact-dependent perturbations of epithelial integrity might result from internalisation of fungal spores. To assess this we first assessed the numbers of internalised wild type and *ΔpacC* spores using a nystatin protection assay (Figure 6A). This revealed that the proportion of wild-type spores internalised by A549 cells ranges from 16 to 23% (Figure 6A). However, epithelial cells were found to internalise *ΔpacC* mutants significantly less avidly than the respective parental isolates (Figure 6A), whereby only ∼10–12% of the initial inoculum had become internalised after 4 hours of co-incubation. At the concentrations used in this assay nystatin exposure was 100% efficient in killing *A. fumigatus* spores (Figure S11).

Given the altered cell wall morphology of *ΔpacC* isolates a plausible explanation for their defective internalisation might include an adhesion defect. We therefore tested the ability of the isolates to adhere to plastic surfaces and epithelial monolayers after 30 minutes of incubation. However, for neither substrate was a difference in adherence observed, either for the *ΔpacC* mutants or wild type isolates (Figure S12). Taken together, these data suggest that internalisation of *A. fumigatus* spores, which is hampered by *pacC* deletion, contributes to contact-dependent monolayer decay.

To assess the role of Dectin-1 in promoting epithelial decay *in vitro* we pre-incubated epithelial cells with the monoclonal anti-Dectin-1 antibody (Mab1859) prior to performing nystatin protection assays. Concordant with a role for Dectin-1 in spore internalisation, epithelial monolayers pre-treated with Mab1859 exhibited a ∼20–30% reduction in internalised spores, relative to untreated epithelia (Figure 6B). As a control we used 0.2 µM cytochalasin D (CD), an inhibitor of actin polymerization, which prevents spore internalisation into A549 cells [18]. The impact of CD treatment (50% reduction) was consistently greater than that of Mab1859, possibly indicating the contribution of additional spore-detecting PRRs driving spore internalisation and/or opsonic phagocytosis and/or incomplete Mab1859-mediated inhibition of Dectin-1 activity.

To assess the impact of spore internalisation upon epithelial integrity, A549 monolayers were pre-treated for 1 hour with CD and detachment after 16 hours was evaluated using our *in vitro* assay. For wild type infections, cellular detachment from epithelial monolayers was significantly reduced from ∼45% to ∼25% in the presence of CD (Figure 6C) but pre-incubation with CD did not alter integrity of *ΔpacC*-challenged monolayers (Figure 6C). These results suggested that actin-mediated internalisation of wild-type spores contributes to epithelial detachment during initial interactions with fungal spores.

To further probe the molecular basis of epithelial decay, monolayers were preincubated with the monoclonal anti-Dectin-1 antibody (Mab1859) prior to co-incubation with *A. fumigatus* spores. Concordant with a damaging role for Dectin-1 mediated internalisation of *A. fumigatus* spores and hyphae in A549 alveolar monolayers, Mab1859 pre-treatment conveyed an almost complete protection of monolayer integrity during co-incubation with wild type *A. fumigatus* isolates (Figure 6C).

To characterise the cell wall defect inhibiting Dectin-1-mediated uptake of *ΔpacC* we examined β-glucan content in the cell walls of wild type and *ΔpacC* spores and hyphae, by incubating fungal cells with a soluble chimeric Dectin-1 Fc protein [45], [46] and quantifying immunofluorescence. This revealed highly anomolous organisation of β-glucan content in *ΔpacC* spores (Figure 6D). Relative to wild type spores which deposit β-glucan at a highly localised, and singular focus of the spore cell wall prior to germination, β-glucan in *ΔpacC* spores adopts a highly diffuse distribution. This phenotype is not a consequence of slowed spore swelling as wild type and *ΔpacC* spores demonstrate equivalent sizes at 4 hours of culture in supplemented DMEM (Figure 6E).

In *Histoplasma capsulatum* α-1,3-glucan promotes virulence by blocking innate immune recognition of β-glucan by Dectin-1. To examine α-1,3-glucan content in *A. fumigatus* spores and hyphae we used immunofluorescence microscopy and anti-α-1,3-glucan antibody, which has previously been used for analysis of the *H. capsulatum* cell wall [47]. Immunofluorescence-mediated detection of this antibody revealed similar distributions of α-glucan in wild type and *ΔpacC* hyphae (Figure S13).

Taken together these data support an important role for internalisation of *A. fumigatus* spores during invasion of the pulmonary epithelium which, in a cell wall-, actin- and Dectin-1 dependent manner permits endocytosis of fungal particles contacting alveolar epithelia.

### Dectin-1 protects murine pulmonary epithelia from *A. fumigatus*-mediated damage

**Figure 7:**
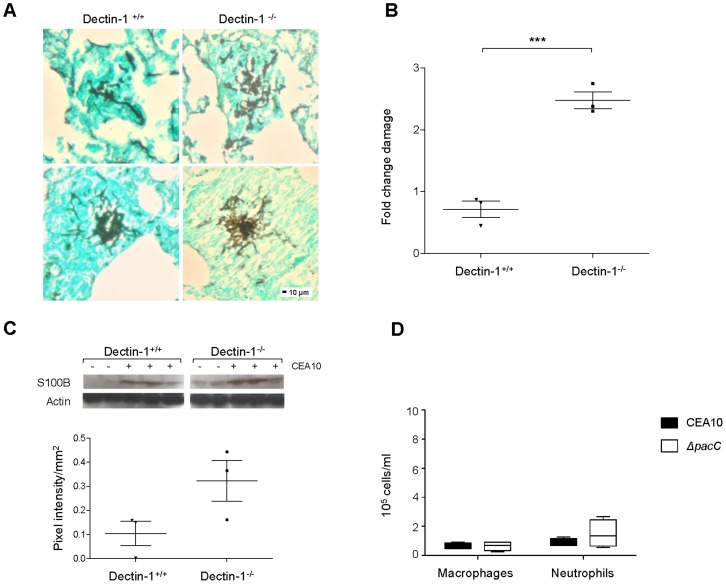
Internalisation and Dectin-1 protect murine pulmonary epithelia from *A. fumigatus*-mediated damage. **(A)** Histopathology of Dectin-1^−/−^ and Dectin-1^+/+^ leukopenic mice (n  =  3) infected with CEA10 (10^8^ spores, 24 hr of infection, GMS and light green staining, 200× magnification). **(B)** LDH/BCA fold change in BALs from Dectin-1^−/−^ and Dectin-1^+/+^ leukopenic mice (n  =  3) infected as in B, technical triplicates. **(C)** S100B western blotting on lung homogenates from Dectin-1^−/−^ and Dectin-1^+/+^ leukopenic mice infected as in B. Densitometry plot where pooled densitometry values for S100B were normalised on pooled densitometry values for actin and background subtracted for the saline controls. **(D)** Macrophages and neutrophils (cells/ml) in BALs from immunocompetent mice infected with *ΔpacC* ^CEA10^ mutant and CEA10 (n  =  4, 10^6^ spores, 24 hr of infection). B and C: unpaired *t* test, *** *p*<0.001 and * 0.01<*p*<0.05.

The extent of epithelial protection afforded by *in vitro* delivery of Mab1859 (Figures 6B and C) was suggestive of a detrimental role for Dectin-1 engagement during *A. fumigatus*-epithelial interactions. To decipher between protective and exacerbatory roles for Dectin-1 in maintenance of epithelial integrity in whole animals, we assessed pulmonary damage after 24 hours of *A. fumigatus* infection in Dectin-1^+/+^ and Dectin-1^−/−^ mice. To study epithelial activities in the absence of confounding leukocyte responses, mice were depleted of leukocytes using a cyclophosphamide and hydrocortisone protocol and lung injury was scored via histological, biochemical and immunoblot assays. In the lungs of Dectin-1^+/+^ and Dectin-1^−/−^ mice, fungal lesions were equivalent in size and invasiveness (Figure 7A) although frequency of fungal lesions was increased in Dectin-1^−/−^ animals (not shown). Epithelial damage was surveyed via quantitation of lactate dehydrogenase (LDH) in BALs (Figure 7B) and analysis of expression of the Dectin-1-independent damage associated molecular pattern (DAMP) protein S100B (Figure 7C), whose major source during *A. fumigatus* infection is epithelial cells [48]. Both assays revealed heightened epithelial damage in the lungs of Dectin-1^−/−^ animals relative to wild type counterparts. Our results indicate that, despite a highly protective role for the anti Dectin-1 antibody Mab1859 during *in vitro* epithelial infections, integrity of Dectin-1 (Figures 7A–C) is essential for limitation of epithelial damage *in vivo*. As neutrophil-depletion and macrophage dysfunction were chemotherapeutically implemented in our murine model we conclude that Dectin-1 activity is essential for protecting the lung epithelium from the damage inflicted by germinating *A. fumigatus* spores.

PacC homologues in the fungal pathogens *C. albicans* and *Cryptococcus neoformans* indirectly modulate interactions with the host interface via governance of fungal cell wall architecture. For both organisms, cell surface defects in Rim101 null mutants appear to be a critical component of altered pathogenicity. In *C. albicans*, oropharyngeal pathogenicity, estimated from *in vitro* assessment of damage in the FaDu cell line, can be partially restored to attenuated Rim101 null mutants via overexpression of the Rim101 target genes *ALS3*, *CHT2, PGA7/RBT6, SKN1* or *ZRT1* [49]. In *C. neoformans* Rim101 null mutants, via cell wall defects, prompt aberrant inflammatory responses, resulting in mild hypervirulence [50]. Thus, in the case of both organisms, host immune responses to altered cell wall composition play a functional role in disease outcome. To test the immunostimulatory capacity of wild-type and *ΔpacC* spores, immunocompetent CD1 mice were infected with 10^6^ spores and the recruitment of macrophages (F4/80^+^) and neutrophils (Ly-6G^+^) to the pulmonary niche was quantified. Relative to mice infected with a wild-type isolate, no significant difference in the number of recruited neutrophils was recorded for mice infected with the *ΔpacC* mutant (Figure 7D). Further, and in stark contrast to findings in *C. neoformans*, we did not observe (in immunocompetent hosts) a hyperinflammatory response to infection with *ΔpacC* mutants. We therefore conclude that anomalous innate immune responses are unlikely to contribute to the altered pathogenicity of *A. fumigatus ΔpacC* mutants. Therefore, in stark contrast to oral and pulmonary infections, respectively with *C. albicans* [49] and *C. neoformans* [50]–[52], modulation of host innate immunity is unlikely to contribute to *A. fumigatus* disease outcome.

Taken together our results indicate that the predominant host-mediated mechanism promoting the non-invasive phenotype of *A. fumigatus ΔpacC* mutants is a failure to engage the Dectin-1 receptor. It is therefore highly likely that *A. fumigatus* exploits this innate immune mechanism to gain entry to the pulmonary epithelium. The important role for Dectin-1 in epithelial protection *in vivo* implies that the full extent of systemic Dectin-1 depletion upon epithelial defences likely extends well beyond defective internalisation of inhaled fungal spores. However, given the propensity of *A. fumigatus* to exploit this mode of tissue entry, it remains possible that the targeted depletion of epithelial Dectin-1 activity would afford protection against invasive, and other, *A. fumigatus* diseases of the lung.

### *ΔpacC* mutants are hypersensitive to cell wall active drugs

**Figure 8:**
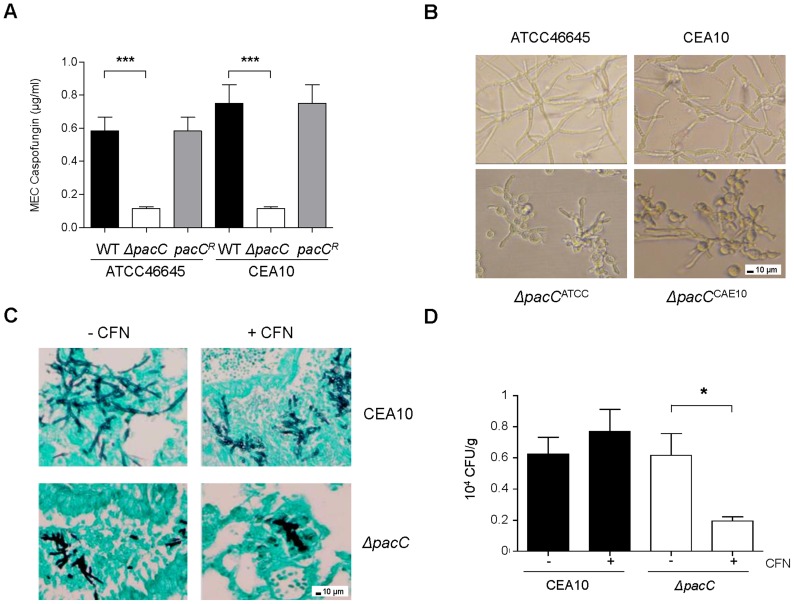
*ΔpacC* mutants are hypersensitive to cell wall active drugs. **(A)** *In vitro* susceptibility testing to caspofungin (CFN). Determination of CFN MEC (μg/ml), twice in triplicates, 1.25 × 10^5^ spores, 48 hr. **(B)** *A. fumigatus* growth in the presence of 16 µg/ml CFN. 400× magnification. **(C)** Histopathology from leukopenic mice infected with CEA10 or *ΔpacC* ^CEA10^ (3 × 10^4^ spores) in the presence or absence of 5 mg/kg CFN. Lungs sections at 24 hr of infection, GMS and light green staining, 200× magnification. **(D)** *In vivo* susceptibility testing to caspofungin. Fungal burden at 48 hr of infection, calculated by CFUs, from leukopenic mice infected as from C. Results are expressed as CFUs per gram of lung tissue. A and D: unpaired *t* test, *** *p*<0.001, **.001<*p*<0.01, and * 0.01<*p*<0.05.

The fungal cell wall is a premier, pathogen-specific target for antifungal drugs. Given the significant cell wall defect observed in *ΔpacC* mutants we predicted altered echinocandin sensitivity relative to wild type isolates. A standard EUCAST assay [53] was used to calculate the susceptibility of isolates, revealing increased susceptibility (Figure 8A) of *ΔpacC* mutants (minimum effective concentration, MEC, of 0.11 µg/ml) compared to that of ATCC46645 (∼ 0.58 µg/ml) and CEA10 (∼ 0.75 µg/ml). *A. fumigatus* strains grown in the presence of 16 µg/ml caspofungin displayed aberrant morphology, elevated branching and shortening of hyphae. Heightened severity of these phenotypes was observed for *ΔpacC* mutants which demonstrated extensive ballooning of hyphal tips (Figure 8B). Given the tendency for chitin increase to promote echinocandin tolerance the heightened susceptibility of *ΔpacC* mutants is surprising; however, an obvious explanation for this effect would be increased porosity due to altered cell wall architecture.

To test the potency of echinocandin agents against pH non-sensing mutants *in vivo* we examined, via viable counts, the effect of caspofungin (5 mg/kg) treatment at 48 hr post-infection. Relative to animals infected with a wild type isolate, fungal burden was significantly decreased in caspofungin-treated mice infected with *ΔpacC* ^CEA10^ (Figure 8C). Histological analysis of lung tissues recovered from mice infected with CEA10 or *ΔpacC* ^CEA10^ in the presence or absence of caspofungin confirmed this observation (Figure 8D). Thus, *ΔpacC* mutants are hypersensitive to the cell wall-active drug caspofungin, a phenotype which extrapolates to mammalian infections (Figures 8C and 8D). Given that the *A. fumigatus* cell wall is essential for viability, agents which selectively inhibit the pH-dependent activation of PacC signalling might provide useful adjuncts to existing antifungal therapies.

## Discussion

Amongst an annual global caseload of 1.5 million fatal mycoses, more than 75% of infections are initiated by inhalation of fungal particles [1]. Despite this, our understanding of the interactions between inhaled fungal pathogens and the respiratory epithelium remains in its infancy. This study addresses disease caused by the major mould pathogen of humans, *A. fumigatus*, and assigns an essential role for the transcription factor PacC in epithelial invasion and pathogenicity. A critical discovery made during this study is the inability of *ΔpacC* mutants to invade the mammalian respiratory epithelium, a hallmark of invasive diseases caused by *A. fumigatus*. Our study confirms that epithelial invasion by this pathogen is a genetically-regulated trait, under PacC regulatory control, and identifies the cellular basis of the deficit to lie with at least two temporally and mechanistically distinct processes, namely protease-mediated monolayer decay and epithelial entry.

Compared to our previous studies of *A. nidulans* pH regulation [29], loss of *A. fumigatus* PacC has a milder impact on alkaline tolerance and *in vivo* germination. At least one mode of essential micronutrient acquisition is different between the two species. In *A. nidulans*, siderophore-mediated iron acquisition is PacC-dependent [54], in *A. fumigatus* it is not (Hubertus Haas personal communication). Certainly this could explain the differences between these two species in germination/growth rates in the mammalian lung.

The *A. fumigatus* genome is predicted to encode more than 100 hydrolytic enzymes [55] some of which are assumed as crucial for liberation of proteinaceous nutrients from host tissues [56], [57]. Early studies found a correlation between high elastinolytic potency of *A. fumigatus* isolates and pathogenicity in mice [58] but a subsequent survey of 73 isolates revealed discordant production of extracellular elastase, acid proteinase and phospholipase amongst strains causing human disease. The elastinolytic neutral metalloprotease Mep (AFUA_8G07080), secreted in *A. fumigatus* culture filtrates and leukopenic murine hosts [59], is dispensable for pathogenicity.

*A. fumigatus* culture filtrates cause epithelial desquamation and destroy F-actin cytoskeletal fibres of *in vitro*-cultured pneumocytes [8], [60]. Kogan *et al.*, 2004 found deletion of the *alp1* gene (AFUA_4G11800) encoding a secreted alkaline protease, or antipain treatment of wild-type culture supernatants to be equally ablative of secreted protease activity *in vitro*. In addition, immunolabelling of the F-actin cytoskeletal fibres of A549 cells revealed that F-actin disruption requires Alp1 integrity. However, an *A. fumigatus* mutant lacking Alp1 retained full virulence in both cortisone-treated [61] and leukopenic mice [15]. Attempts to implement wholesale depletion of *A. fumigatus* protease expression have also failed to unearth avirulent mutants. A doubly protease-deficient mutant lacking Alp1 and Mep, which is completely deficient in collagenic proteolytic activity at neutral pH *in vitro*, is fully virulent in a cortisone-treated murine model [13]. Furthermore, an *A. fumigatus ΔprtT* mutant lacking a conserved positive regulator of secreted proteases suffers a 70% reduction in casein proteolytic activity, but is also fully virulent in leukopenic mice. These findings cast doubt upon the true relevance of protease production to *A. fumigatus* pathogenicity [10]. Comparison of the PacC and PrtT [9] regulons revealed 83 and 31 secreted gene products as being down-regulated by PacC and PrtT (AFUA_4G10120) respectively, amongst which only 8 are commonly down-regulated (Dataset S3). Given the fully virulent phenotype of the *ΔprtT*, mutant we can confidently surmise that none of these gene products, acting alone or in combination with each other, can support tissue-invasive growth in the mammalian host. Thus their aberrant expression in the *ΔpacC* mutant is unlikely to explain its non-invasive phenotype. Concordant with this conclusion, and with the fully virulent phenotype of a *Δmep;Δalp1* mutant [13], the inclusion of genes encoding both Alp1 and Mep1 (Dataset S3) amongst those down-regulated in both PacC and PrtT null mutants cannot, alone, explain the non-invasive phenotype of PacC null mutants. Loss of PacC negatively impacts a further 75 uncharacterised secreted gene products. If the tissue invasive growth of *A. fumigatus* is solely proteolytically mediated, we predict that important enzymatic functions will be amongst them. Investigation of this hypothesis lies beyond the scope of this study but is an ongoing component of our further study, as is the co-operative activity of fungal protease- and toxin-mediated assaults upon the mammalian pulmonary epithelium.

The finding that epithelial monolayers are resistant to culture filtrates obtained from earlier (16 hour) time points of fungal growth highlights the existence of at least one additional, and earlier acting, perturbation of host tissue. Soluble effectors of epithelial detachment are not immediately secreted by metabolically active *A. fumigatus* spores and a phased mode of *A. fumigatus* assault, commencing with contact-dependent perturbation, is likely responsible for monolayer perturbation. In agreement with this hypothesis, we found cytochalasin D-mediated inhibition of actin polymerisation to be partially protective of epithelial monolayer integrity, and *ΔpacC* spores to be far less avidly internalised than wild-type counterparts. Amongst the repertoire of invasion tactics employed by fungal pathogens at host epithelia, induced endocytosis, active penetration and participation of host factors have been implicated [52]. The results of our study implicate all three activities as having relevance to the host-*A. fumigatus* interaction, but with several critical differences relative to studies of other fungal pathogens. Firstly, the occurrence of induced endocytosis, as evidenced by sensitivity of the internalisation process to cytochalasin D-mediated inhibition (Figure 6C), promotes epithelial disintegration by both live and dead fungal elements. This finding stands in stark contrast to epithelial invasion by *C. albicans* where internalisation of live germinated cells, but not killed cells, leads to host damage [62]. Second, the existence of *A. fumigatus* invasins remains thus far unproven. Certainly, from bioinformatics analyses, evidence for highly conserved homologues for the *C. albicans* invasin Ssa1 can be gleaned. However the expression of this homologue is not impacted by *pac*C gene deletion and *A. fumigatus* lacks any homologue of the Als3 invasin altogether [63], [64]. Our finding that cell wall extracts can impact epithelial integrity, and the relevance of Dectin-1 to this process, implies the existence of an invasin-independent mode of epithelial entry for *A. fumigatus*. In our studies cytochalasin D imposed an incomplete block upon spore internalisation suggesting that at least a subset of fungal elements can access the internal environment of epithelial cells via an ‘active penetration’ mechanism, as recently documented for *C. albicans* [65]. These important differences in the way in which different fungal pathogens interact with physiologically distinct epithelia highlight the current paucity of information on fungal interactions with pulmonary epithelia and, given that the vast majority of invasive mycoses are initiated via inhalation of fungal particles [1], should prompt renewed scrutiny of fungal interactions with mammalian lung tissues.

The mechanism by which epithelial cells recognise and internalise *A. fumigatus* conidia remains poorly characterised, as does the relevance of such activity to disease outcome. Our study demonstrates that uptake of *A. fumigatus* spores, by type II pneumocytes, is dependent upon a) actin polymerisation b) fungal cell wall/surface composition c) integrity of PacC and d) the β-1,3-glucan receptor Dectin-1, and moreover, that such interactions can negatively impact epithelial integrity. Dectin-1 expression in non-myeloid cells is increasingly frequently reported and has demonstrated relevance in β-1,3-glucan-(curdlan) exposed bronchiolar and alveolar type II cells [43], poly IC challenge of human bronchial epithelial cells [66], *Mycobacterium ulcerans* infection of epidermal keratinocytes and *Mycobacterium tuberculosis* infection of A549 epithelia [67], [68]. The A549 cell surface constitutively expresses Dectin-1, regardless of infection by *A. fumigatu*s [44]. In humans, a Y238X Dectin-1 polymorphism is a risk factor for invasive aspergillosis in haematopoietic stem cell recipients. Cunha et al. (2010) found, in experimental HSCT, that transplant of stem cells from Dectin-1^-^ donors to wild-type recipients resulted in lessened susceptibility to invasive aspergillosis. Thus by restricting the Dectin-1 deficiency to cells of myeloid origin, susceptibility to invasive aspergillosis was not altered. We found Dectin-1 deficiency, in the absence of leukocytes, to heighten epithelial damage in the whole animal host relative to leukopenic wild-type animals. This observation and several of our findings support a curative role for Dectin-1 mediated internalisation of *A. fumigatus* spores. According to Han *et al*., 2011, internalisation of *A. fumigatus* by A549 epithelial cells can be correlated with membrane phosphatidylcholine cleavage, a process which is closely linked to alteration of cytoskeletal actin dynamics, and prompted by exposure to β-1,3-glucan. This finding is consistent with our observation that cell wall extracts and killed *A. fumigatus* hyphae can perturb epithelial integrity. Our data, and those of Chaudhary *et al*., 2012, who found that bronchial ECs from cystic fibrosis sufferers demonstrate impaired uptake and killing of conidia, are highly suggestive of a curative role for EC activities during exposure to *A. fumigatus* spores. It does, however, remain feasible that the pulmonary epithelium provides a reservoir for *A. fumigatus* spores, and the attenuated phenotype of the *ΔpacC* mutant is consistent with such a theory. It also remains feasible that contact-dependent perturbation of epithelia facilitates subsequent protease-mediated damage by exposing subepithelial structures and facilitating fungal adhesion. Studies of *C. albicans* have revealed several means of fungal entry into epithelial cells, including self-induced endocytosis, and protease-mediated decay, the latter impacting, via Rim101-mediated E-cadherin degradation, the disintegration of oral epithelia [69]. It is most likely that multiple mechanisms contribute also to the pathogenicity of *A. fumigatus*. What is unique about PacC in *A. fumigatus*, is the critical role played by PacC-dependent factors in all of these processes and the profound requirement for PacC to orchestrate epithelial entry, protease-mediated epithelial decay, invasive growth and pathogenicity. These findings not only identify PacC as a critical master regulator of pathogenicity determinants in *A. fumigatus*, but also heighten its relevance as an antifungal target.

## Materials and Methods

### Fungal strains, media and treatments

*A. fumigatus* strains used in this study are listed in Table S1. *A. fumigatus* strains were cultured at 37°C in *Aspergillus* Minimal Media (AMM) or *Aspergillus* Complete Media (ACM) [70]. For preparation of *A. fumigatus* culture supernatants, 10^6^ spores/ml were grown in Dulbecco's Modified Eagle Medium (DMEM, Sigma) supplemented with 10% foetal bovine serum (FBS, Sigma) and 10% penicillin and streptomycin (Sigma) at 37°C, 5% CO_2_ for either 16, 48, 60 or 72 hr. Supernatants were doubly filtrated through Miracloth and centrifuged for 10 min at 4000 rpm to remove any hyphal fragments. To inhibit protease activity in culture supernatants, filtrates were treated with the serine and cysteine protease inhibitor antipain (10 µg/ml) for 1 hr. For preparation of *A. fumigatus* cell wall extracts, see Text S1. For analyses of cell wall composition, *A. fumigatus* strains were harvested from an ACM plate (0 hr) as previously described or from 10^5^ spores/ml cultures grown in AMM broth for 4, 8 and 16 hr at 37°C with shaking at 200 rpm. For *in vitro* challenge of epithelial monolayers with killed hyphae 10^4^ spores/ml were incubated in supplemented DMEM at 37°C, 5% CO_2_ for 18 hr (parental isolates and reconstituted strains) or 36 hr (*ΔpacC* mutants). *A. fumigatus* hyphae were killed by incubating them in PBS supplemented with 0.02% thimerosal (Sigma) overnight at 4°C [71], a treatment which preserves cellular integrity. Before incubation with monolayers, killed hyphae were washed twice in PBS. Killing was verified by plating a 1∶100 dilution of killed hyphae in ACM plates.

### Generation of *ΔpacC* mutants

*ΔpacC* mutants in the genetic backgrounds CEA10 (*ΔpacC* ^CEA10^) and ATCC46645 (*ΔpacC* ^ATCC^) were constructed by gene replacement (Figure S1A) using a split-marker strategy [72]. For details, see Text S1. Initial phenotypic analyses, including survival analyses were performed using both *ΔpacC* ^ATCC^ and *ΔpacC* ^CEA10^ mutants, while subsequent analyses of gene expression were limited to the *ΔpacC* ^ATCC^ mutant. For analyses of *ΔpacC* fungal burden in mice we opted to use the lesser attenuated *ΔpacC* ^CEA10^ to prolong fungal occupancy of the murine lung.

### *Aspergillus* phenotypic analysis

Dilutions of 10^3^ conidia were inoculated onto ACM or supplemented AMM (as shown on Table S3) and incubated for 48 hr and 72 hr respectively. Images were captured using a Nikon Coolpix 990 digital camera.

### Electromobility shift assays

Spores were inoculated at a density of 1×10^6^ to 2×10^6^ spores/ml, in 100 ml of liquid ACM, and grown for 16 hr at 37°C. Next, media was buffered to pH 5.0 with 100 mM glycolic acid pH 5.0 or to pH 8.0 with 100 mM Tris-HCl pH 8.0, pH shifts were performed for 1 hour. 10 mg of protein was extracted from washed mycelia as described previously [73]. Protein concentrations were determined using the Bradford assay [74]. The *ipnA2* probe was synthesised and labelled as described previously [27], [73]. Densitometry data were obtained by measuring pixel intensity/mm^2^ for the relevant bands using a Phosphorimager FLA-3000 (FujiFilm) and Multi-Gauge V3.0 software.

### Murine infections

Murine infections were performed under UK Home Office Project Licence PPL/70/6487 in dedicated facilities at Imperial College London. For all experiments *A. fumigatus* spores were harvested and prepared as previously described [75] and viable counts from administered inocula were determined, following serial dilution, by growth for 24–48 hr on ACM. Mice were housed in individually vented cages and anaesthetized by halothane inhalation and infected by intranasal instillation of spore suspensions. Mice were rendered leukopenic by administration of cyclophosphamide (150 mg/kg, intraperitoneal) on days −3, −1, +2 and every subsequent third day, and a single subcutaneous dose of hydrocortisone acetate (112.5 mg/kg) administered on day −1.

**Survival analysis**. Leukopenic male CD1 mice (18–22 g) were infected by intranasal instillation of 5.0 × 10^4^ or 6.0 × 10^5^ conidia in 40 µl of saline solution. Mice were weighed every 24 hr from day of infection and visual inspection made twice daily. In the majority of cases the end-point for survival experimentation was a 20% reduction in body weight measured from day of infection, at which point mice were sacrificed.

**Histological and transcriptomic analyses**. Leukopenic male CD1 mice (n  =  8) were infected with 10^8^ conidia in 40 µl of saline solution. At the relevant time-points post-infection, mice were sacrificed and lungs were partitioned, using surgical sutures, into lobes destined for transcriptomic or histological analysis. Bronchoalveolar lavages (BALs) were performed using three 0.5 ml aliquots of pre-warmed sterile saline. BALs were snap frozen immediately following harvesting using liquid nitrogen. Lobes for histological sectioning were removed and immediately fixed in 4% (v/v) formaldehyde (Sigma). Lungs were embedded in paraffin prior to sectioning and stained with haematoxylin and eosin or light green and Grocott's Methenamine Silver. Images were taken using a Reichert-Jung (Slough, UK) Polyvar microscope using brightfield illumination at 40X magnification.

**Fungal burden analyses**. Immunosuppressed male CD1 mice (n  =  5) were infected with 3.75 × 10^5^ spores. Mice were culled and whole lungs were collected after 24 and 48 hr of infection.

#### Antifungal drug susceptibility

To test susceptibility to caspofungin, leukopenic, male CD1 mice (n  =  5) were infected with 3 × 10^4^ spores in 40 µl of saline solution and treated with 5 mg/kg caspofungin by intraperitoneal injection on days −3, −1 and +1. Lungs were homogenised, serially diluted and cultured in duplicate in a serial dilution series of up to 10^−4^, on ACM.

#### Cellular content of BALs

Immunocompetent male CD1 mice were infected with 10^6^ spores in 40 µl of saline solution. Mice were culled after 24 hr and BALs were collected using three 0.5 ml aliquots of sterile saline.

#### Assessment of epithelial damage

Leukopenic male CD1 and C57BL/6 Dectin-1^−/−^ [76] were infected with 10^6^ spores in 40 µl of saline solution and culled after 24 hr of infection. Lungs were collected for histopathology and western blotting. BALs, collected using three 0.5 ml aliquots of pre-warmed sterile saline, were used for LDH quantification.

### Temporal analyses of *A. fumigatus* gene expression during murine infection

BAL samples were centrifuged at 14000 rpm for 5 min and the pellet was washed with 500 µl ice cold H_2_O to lyse host cells. Seven BALs were pooled, resuspended in 450 µl ME-RLC buffer (QIAGEN) and ground in liquid nitrogen with a pestle and mortar. RNA was then extracted using RNeasy Kit (QIAGEN). A reference RNA sample was extracted from *A. fumigatus* ATCC46645 conidia harvested from an ACM plate. Conidia were washed thoroughly with sterile water, quickly frozen in liquid nitrogen, and disrupted by grinding. Total RNA was extracted using RNeasy Kit (QIAGEN). The quality of RNA used for microarray analysis was checked using a Nanodrop ND-1000 Spectrophotometer (Nanodrop, Wilmington, USA). Only RNA with an A_260_/_280_ and an A_260_/_230_ ratio> 1.9 was used for the experiments. Labelled cDNA samples were synthesised as described previously [30]. Protocols for direct labelling and hybridisation of cDNA probes can be found on the JCVI website (http://pfgrc.jcvi.org/index.php/microarray/protocols.html). The *A. fumigatus* oligonucleotide slides version 3 was used for microarray hybridization (http://pfgrc.jcvi.org/index.php/microarray/array_description/aspergillus_fumigatus/version3.html).The phase- or strain-specific comparative analysis of gene expression datasets was conducted in Genespring GX 11.02 (Agilent). Normalised log_2_ expression ratios were filtered on expression level and differentially regulated transcripts were defined as having log_2_ (Cy5/Cy3) greater than the arbitrary thresholds of ± 1.5. Raw data have been deposited in the Gene Expression Omnibus (GEO) (http://www.ncbi.nlm.nih.gov/geo/) under accession number GSE54810. Functional analysis of differentially-expressed gene cohorts was implemented by DAVID (http://david.abcc.ncifcrf.gov/) [77], [78]. Microarray data was validated by qPCR as described in Text S1.

### Analysis of A549 epithelial monolayer integrity

Human pulmonary carcinoma epithelial cell line A549 (American type culture collection, CCL-185) was used throughout this study. For all experiments, cells were maintained at 37°C, 5% CO_2_ in supplemented DMEM. Epithelial cells were used after the second or third passage. For all experiments, 10^5^ A549 cells were seeded in 6-well tissue culture plates and incubated to ≥ 90% confluence. Monolayers were challenged with 10^5^ spores/ml, 200 µl of supernatant or 200 µl of cell wall extract. Following co-incubation with *A. fumigatus* spores, cell wall extracts or supernatants, monolayers were washed 3 times with PBS and adherent A549 cells were counted in 3 fields of view at magnifications of 20 or 40 (Nikon Eclipse TS100). Washing was omitted for analyses of contact-dependent damage.

### ^51^chromium release assay

Damage to A549 epithelial cells by the various strains of *A. fumigatus* was determined using a previously described method at 16, 20 and 24 hr of co-incubation [35]. The ^51^Cr content of the medium and lysates was measured and the degree of epithelial cell damage was calculated and corrected for spontaneous chromium release by uninfected epithelial cells.

### *A. fumigatus* cell wall analysis

#### Live-cell microscopy

Fungal cell walls were visualized using the chitin binding dye calcofluor white (Sigma). 10^5^ conidia were grown in 8-well slide culture chambers in AMM (pH 6.5) at 28°C for 17–18 hr and stained with 10 µg/ml of calcofluor white for 5 min prior imaging on a Nikon Eclipse TE2000E microscope with DIC optics, a 60× (1.3 NA) plan fluor objective, and equipped with an ORCA-ER CCD camera (Hamamatsu, Welwyn Garden City, UK) driven by the MetaMorph NX1.1 software for image acquisition. For calcofluor white, a Nikon UV-2A filter cube (excitation filter 355/15 nm BP, dichroic mirror 400 nm LP, emission filter 420 nm LP) was used. Images were processed and analysed using the software Image J version 1.47. Images show maximum intensity projections with inverted look-up tables (LUTs).

#### Electron microscopy

Preparation of samples for Transmission Electron Microscopy (TEM) were performed as previously described [79]. See Text S1.

#### Preparation of cell wall extracts for challenge of monolayer integrity and composition analysis

Cell walls were extracted with some modifications as described previously [80], [81]. See Text S1.

### Immunofluorescence

To analyse the localisation of α-1,3-glucan or β-1,3-glucan on *A. fumigatus* cell walls, isolates (10^5^ or 10^4^ spores/ml) were grown in 8-well slide culture chambers (Nalge Nunc International, Rochester, NY) in supplemented DMEM for 4, 8, 12 or 16 hr. α-1,3-glucan was visualized using 0.1 mg/ml mouse IgMγ MOPC-104E (Sigma, in PBS buffer) as primary antibody and 0.1 mg/ml Alexa Fluor 488 goat anti-mouse IgM (μ chain) antibody (Life technologies, in PBS buffer) [47], [82], [83]. β-1,3-glucan was visualized 5 µg/ml Fc-dectin-1 fusion (kind gift from Dr G.D. Brown, University of Aberdeen) coupled with 15 µg/ml goat anti-human IgG (H+L) Fluorescein conjugated antibody [45], [46]. Briefly, samples were incubated with primary antibodies for 30 minutes, before incubation with the secondary antibodies for 30 minutes in the dark.

To visualise epithelial monolayers co-incubated with *A. fumigatus* strains, A549 cells were seeded in 2-well slide culture chambers (Nalge Nunc International, Rochester, NY) in supplemented DMEM. At 90% confluence, epithelial monolayers were incubated with *A. fumigatus* isolates (10^5^ spores/ml) for 16 hr. After washing the monolayers three times with PBS, samples were incubated in supplemented DMEM with 10 µg/ml FITC-labelled concanavalin A (Molecular Probes) and 0.4 mg/ml calcofluor white (Sigma), to visualize respectively epithelial cells and hyphae. Labelling was performed for 30 minutes at 37°C, 5% CO_2_.

After rinsing with PBS, samples were imaged using a Nikon Eclipse TE2000E microscope with DIC optics, a 20× plan fluor objective or 60× (1.3 NA) plan fluor objective, and equipped with an ORCA-ER CCD camera (Hamamatsu, Welwyn Garden City, UK) driven by the MetaMorph NX1.1 software for image acquisition. For Alexa Fluor 488, FITC and fluorescein, a Nikon B-2A filter cube (excitation filter 470/20 nm BP, dichroic mirror 500 nm LP, emission filter 515 nm LP) was used. For calcofluor white, a Nikon UV-2A filter cube (excitation filter 355/15 nm BP, dichroic mirror 400 nm LP, emission filter 420 nm LP) was used. Images were processed and analysed using the software Image J version 1.47.

### Adhesion assay

Adhesion was tested using a modification of the protocol in Gravelat *et al.*, 2012 [84] as described in Text S1.

### Internalisation of *A. fumigatus* spores by A549 epithelia

#### Pretreatment of epithelia

For evaluation of detachment or nystatin protection assay, epithelial monolayers were pre-treated for 1 hour with 0.2 µM cytochalasin D (CD, Sigma, resuspended in DMSO) or 0.3 µg/ml α-Dectin-1 antibody (R & D) or 0.3 µg/ml Mouse IgG2B Isotype Control (Clone 20116) (R & D).

#### Nystatin protection assay

A549 density was checked by enumeration, and epithelial monolayers were infected with 10^6^ spores/ml. Viable counts of inocula were determined following serial dilution by plating on ACM and growth for 24–48 hr. The nystatin protection assay was performed as in Wasylnka *et al.*, 2002 [18] allowing spore internalisation for 4 hr. The percentage of internalisation was calculated relative to the total amount of conidia inoculated and applying a correction for an MOI of 1. Experiments were performed in technical and biological duplicate for wild-types and triplicate for *ΔpacC* mutants. Susceptibility of the strains to nystatin was verified as described in Text S1.

### LDH assay

The release of lactate dehydrogenase (LDH) was assessed in BALs using the Cytox 96 Non-Radioactive Cytotoxicity Assay kit (Promega) according to manufacturer's instructions. BAL samples were assessed in triplicate and averaged values were normalised to the total amount of protein as measured in triplicate using a bicinchoninic acid assay (BCA) assay (Sigma) according to manufacturer's instructions.

### Western blotting

Lungs were homogenised in 1 ml of PBS (pH 7.4) containing protease inhibitor cocktail (Roche) and protein concentration was measured by BCA, using a BSA as standard (Sigma). 9 µg of protein was analysed by western blotting [85]. A 1∶1000 dilution of a α-S100B antibody (Abcam) was used, in parallel with an α-actin antibody (Cell Signaling) for normalisation of loading.

### FACS analysis of BALs

BALs were collected using 3 ml of PBS and a further 5 ml of PBS were added at the time of preparation of the samples for FACS analysis. Cell pellets were resuspended in 1 ml red blood lysis buffer (Sigma). Blocking of the Fc receptor to remove unspecific signal was achieved by incubating the samples with 0.5 µg of an anti-Mouse CD16/CD32 antibody (E-bioscience) in 100 µl of 0.1% BSA PBS. 14 µl of antibody mix was added for labelling of macrophages (α-F4/80-APC-Cy7, 5 µl, Biolegend), leukocytes (α-CD45-PE, 2 µl, E-bioscience) and neutrophils (α-Ly-6G-BV421, 2 µl, Biolegend). Samples was analysed using a BD Fortessa cell analyser. Data acquisition and analysis were performed using respectively the software Diva and FlowJo. For each sample (n  =  4, plus 2 controls), cell population size for macrophages (F4/80^+^) and neutrophils (Ly-6G^+^) were expressed as cells/ml.

### Antifungal susceptibility

*In vitro* susceptibility testing of *A. fumigatus* strains was performed according to the European Committee for Antimicrobial Susceptibility testing (EUCAST) standard method [53]. Caspofungin was tested on *A. fumigatus* strains in biological and technical triplicate. 1.25 × 10^5^ strains were grown with RPMI1640, 0.165 mol/L MOPS, pH 7.0 and incubated at 37°C for 48 hr. The final concentration of caspofungin tested ranged from 0.03 to 16 ug/ml.

### Statistical analysis of data

GraphPad Prism was used to interpret data and *p* values were calculated through Log Rank analysis (for comparative survival), unpaired *t* tests or 1-way ANOVA tests as indicated. Error bars show the Standard Error of the Mean (SEM). ***p<0.001, 0.001 <**p<0.01, and 0.01 <*p<0.05

### Online supporting information

Figure S1 shows the strategies for construction and validation of *A. fumigatus ΔpacC* mutants. Figure S2 shows growth of *A. fumigatus* isolates on laboratory culture media, as images and as radial growth rates normalised to pH 6.5. Figure S3 shows pH-dependency of PacC processing, as measured using EMSA analyses. Figure S4 shows the experimental set-up and outputs of in-host transcriptomic analysis of *A. fumigatus* wild-type and *ΔpacC* activities. Figure S5 shows a heat map of differentially expressed *A. fumigatus* gene products having predicted signal peptides. Figure S6 shows a heat map of differentially expressed *A. fumigatus* gene products having putative or demonstrated roles in cell wall biosynthesis. Fig. S7 shows a heat map of differentially expressed *A. fumigatus* gene products involved in gliotoxin biosynthesis (AFUA_6G09570-AFUA_6G09740). Figure S8 shows qPCR validation of microarray data. Figure S9 shows analysis of *A. fumigatus* protease activity using a qualitative gelatine degradation assay. Figure S10 shows the electron microscopy of *A. fumigatus ΔpacC* ^ATCC^ mutant and the respective parental isolate at 0, 4, 8 and 16 hr of growth. Figure S11 shows equivalent nystatin-mediated killing of wild type and mutant isolates used in this study. Figure S12 shows equivalent adhesion of mutant and wild types isolates to plastic, and to epithelia *in vitro*. Figure S13 shows immunofluorescence analysis of α-glucan distribution in *A. fumigatus* germlings. Table S1 and S2 list the *A. fumigatus* strains and oligonucleotides used in this study respectively. Table S3 lists the *A. fumigatus* phenotypic testing conditions tested. Dataset S1 shows the temporal analysis of *A. fumigatus* gene expression during intiation of murine pulmonary aspergillosis. Dataset S2 shows the genes differentially regulated, relative to wild type, in a host-infecting *A. fumigatus ΔpacC* ^ATCC^ mutant. Dataset S3 shows the expression of genes encoding secreted gene products which are regulated by either PrtT [9] or PacC, or both transcription factors. Text S1 contains the supplementary material and methods.

### Accession numbers

The genes and gene products (with accession numbers at http://www.cadre-genomes.org.uk/) studied in this work is *pacC* (AFUA_3G11970). Also mentioned in the text are *prtT* (AFUA_4G10120), *mep* (AFUA_8G07080), *alp1* (AFUA_4G11800), *β-tubulin* (AFUA_7G00250) and the gliotoxin cluster (AFUA_6G09570-AFUA_6G09740).

## Supporting Information

Dataset S1 **Temporal analysis of** ***A. fumigatus*** **gene expression during initiation of murine pulmonary aspergillosis.** Differentially-expressed genes (log_2_ ratios ≥ +/− 1.5 relative to ungerminated spores) were categorised as INVARIANT UP/DOWN (differentially expressed at all time points of analysis), EARLY UP/DOWN (differentially expressed at any or all of 4, 8 and 12 hours post-infection) and LATE UP/DOWN (differentially expressed at either or both of 12 and 16 hours post-infection). Functional analysis of differentially-expressed gene cohorts was implemented by DAVID (http://david.abcc.ncifcrf.gov/).
(XLSX)

Dataset S2 **Genes differentially regulated, relative to wild type, in a host-infecting** ***A. fumigatus ΔpacC*** **^ATCC^ mutant.** Differentially-expressed genes (log_2_ ratios ≥ +/− 1.5 relative to wild type) were categorised as ALWAYS UP/DOWN or UP/DOWN at ≥ one time point of infection. Functional analysis of differentially-expressed gene cohorts was implemented by DAVID (http://david.abcc.ncifcrf.gov/).
(XLSX)

Dataset S3 **Expression of genes encoding secreted gene products which are regulated by either PrtT or PacC, or both transcription factors.**
(XLSX)

Figure S1 **Construction and validation of** ***A. fumigatus ΔpacC*** **mutants.** Schematic for the construction of *A. fumigatus ΔpacC* mutants by gene replacement using a split pyrithiamine resistance marker **(A)** and Southern blot analysis of *A. fumigatus* wild-type and *ΔpacC* mutants **(B)**. According to the first strategy employed, genomic DNA on indicated strains was digested with SalI and probed with two hybridisation probes generated using the oligonucleotides PacCSB1 & PacCSB2 (Probe 1) and LucPtrAF & LucPtrAR (Probe 3) (Table S2). The expected signals for Probe 1 were 7883 bp for the wild-type and 3355 bp for the *ΔpacC* mutants. For Probe 3, no signal was expected for the wild-type, whereas for a single, homologous integration of the deletion cassette the expected signal was 3355 bp. According to the second strategy employed, genomic DNA of indicated strains was digested with EcoRV and probed with a hybridisation probe generated using oligonucleotides opacC3 and opacC6 (Probe 2) (Table S2). The expected signals for Probe 3 were 3709 bp for the wild-type and 3501 bp for the *ΔpacC* mutants.
(TIFF)

Figure S2 **Colonial growth of wild type and** ***ΔpacC*** **mutants.** **(A)** Colonial growth phenotypes on ACM, 48 hr of growth, 10^3^ conidia. **(B)** Percent radial growth at pH 8.0 relative to pH 6.5, 72 hr of growth, 10^3^ conidia, n  =  3, unpaired *t* test, *** *p*<0.001 and ** 0.001 <*p*<0.01. **(C)** Percent radial growth at pH 7.2 relative to pH 6.5, 72 hr of growth, 10^3^ conidia, n  =  3.
(TIFF)

Figure S3 **Processing of** ***A. fumigatus*** **PacC.** **(A)** PacC processing after shift to acidic (pH 5.0) and alkaline (pH 8.0) by EMSA. Low mobility and high mobility forms of PacC are indicated on the right side. **(B)** Densitometry plot of EMSA data expressed, per complex, as a function of total PacC protein.
(TIFF)

Figure S4 **In-host transcriptomic analysis of** ***A. fumigatus*** **wild-type and** ***ΔpacC*** **activities.** **(A)** Experimental design depicting hybridisation schemes. (I) Analysis of wild-type temporal transcription profile during initiation of murine infection comparing gene expression at 4 independent time points (4, 8, 12 and 16 hr) to a common RNA reference extracted from wild-type spores. (II) Analysis of *ΔpacC* temporal transcription profile during initiation of murine infection comparing gene expression at 4 independent time points (4, 8, 12 and 16 hr) to a common RNA reference extracted from *ΔpacC* spores. (III) Comparative, stage-specific, analysis of *ΔpacC* and wild-type transcript profiles during initiation of murine infection (4, 8, 12 and 16 hr). **(B, C, D)** Up- and down-regulated genes (cut-off ± 1.5) for the wild-type (B), *ΔpacC* (C) and comparative (D) time courses. Venn diagrams depict the numbers of genes differentially regulated at each time point of the analyses. Profile plots indicate temporal behaviours of all differentially regulated genes relative to the 4 hr time point.
(TIFF)

Figure S5 **Heat-map depiction of temporal in-host gene expression for wild type and** ***ΔpacC*** **.** Heat maps depict results from time-series infection studies and profile gene expression for predicted secreted proteins. Colouration indicates magnitude of log_2_ ratio relative to RNA reference (ungerminated spores).
(TIFF)

Figure S6 **Heat-map depiction of temporal in-host gene expression for wild type and** ***ΔpacC*** **.** Heat maps depict results from time-series infection studies and profile gene expression for predicted cell wall biosynthetic gene products. Colouration indicates magnitude of log_2_ ratio relative to RNA reference (ungerminated spores).
(TIF)

Figure S7 **Heat-map depiction of temporal in-host gene expression for wild type and** ***ΔpacC*** **.** Heat maps depict results from time-series infection studies and profile gene expression for genes (AFUA_6G09570-AFUA_6G09740; AFUA_6G09745 omitted from the microarray) of the gliotoxin cluster. Colouration indicates magnitude of log_2_ ratio relative to RNA reference (ungerminated spores).
(TIF)

Figure S8 **qPCR validation of microarray data.** Accuracy of microarray data was independently verified by quantitative RT-PCR on selected transcripts, values **(A)** and graphical representation **(B)**. Oligonucleotides used for this analysis are detailed in Table S2. Fold change due to treatment (-1/ΔC_T_) was calculated using *A. fumigatus* Act1 (AFUA_6G04740) as a house-keeping gene.
(TIFF)

Figure S9 **Qualitative analysis of** ***A. fumigatus*** **protease activity in cell culture filtrates.** Culture supernatant (50 µl of supplemented DMEM + A549 cell culture, 10^6^ spores/ml) was spotted onto X-ray film, incubated at 42°C and washed after overnight digestion with water. Positive control is represented by trypsin (50 BAEE units). Trypsin hydrolyzes N-benzoyl-L-arginine ethyl ester (BAEE) and one BAEE unit equals the amount of the enzyme determining an increase in absorbance of 0.001 per minute at 25°C and 253 nm.
(TIFF)

Figure S10 **Electron microscopy of** ***A. fumigatus ΔpacC*** **^ATCC^ mutant and the respective parental isolate at 0, 4, 8 and 16 hr of growth.**
(TIF)

Figure S11 **Nystatin-mediated killing of wild type and** ***ΔpacC*** **mutant spores.**
(TIFF)

Figure S12 **Adhesion of wild type and** ***ΔpacC*** **mutant spores to plastic and A549 monolayers.**
(TIFF)

Figure S13 **Immunofluorescence-mediated imaging of** ***A. fumigatus*** **α-glucan.**
(TIF)

Table S1 ***A. fumigatus* strains used in this study.**
(DOCX)

Table S2 **Oligonucleotides used in this study.**
(DOCX)

Table S3 ***A. fumigatus* phenotypic testing.**
(DOCX)

Text S1 **Supplementary material and methods.**
(DOCX)
