# Correction: The pH-Responsive PacC Transcription Factor of *Aspergillus fumigatus* Governs Epithelial Entry and Tissue Invasion during Pulmonary Aspergillosis

**DOI:** 10.1371/journal.ppat.1004802

**Published:** 2015-04-08

**Authors:** The PLOS Pathogens Staff

The concentration of α-Dectin-1 antibody and isotype control antibody is incorrect throughout the manuscript. The correct concentration of α-Dectin-1 antibody and isotype control antibody is 3 μg/ml.
