# Correction: The pH-Responsive PacC Transcription Factor of *Aspergillus fumigatus* Governs Epithelial Entry and Tissue Invasion during Pulmonary Aspergillosis

**DOI:** 10.1371/journal.ppat.1004943

**Published:** 2015-06-18

**Authors:** Margherita Bertuzzi, Markus Schrettl, Laura Alcazar-Fuoli, Timothy C. Cairns, Alberto Muñoz, Louise A. Walker, Susanne Herbst, Maryam Safari, Angela M. Cheverton, Dan Chen, Hong Liu, Shinobu Saijo, Natalie D. Fedorova, Darius Armstrong-James, Carol A. Munro, Nick D. Read, Scott G. Filler, Eduardo A. Espeso, William C. Nierman, Hubertus Haas, Elaine M. Bignell

The authors would like to correct the following sentence in the Materials and Methods section of the article:

“For preparation of A. fumigatus culture supernatants, 10^6^ spores/ml were grown in Dulbecco’s Modified Eagle Medium (DMEM, Sigma) supplemented with 10% foetal bovine serum (FBS, Sigma) and 10% penicillin and streptomycin (Sigma) at 37uC, 5% CO2 for either 16, 48, 60 or 72 hr.”

In this study, Dulbecco's Modified Eagle Medium (DMEM, Sigma) was supplemented with 10% foetal bovine serum (FBS, Sigma) and 1% penicillin and streptomycin (Sigma), not 10% penicillin and streptomycin (Sigma) as originally indicated.
