# Supplementary figures and images for: The pH-Responsive PacC Transcription Factor of Aspergillus fumigatus Governs Epithelial Entry and Tissue Invasion during Pulmonary Aspergillosis
Source: PLoS Pathog. 2014 Oct 16;10(10):e1004413. doi: 10.1371/journal.ppat.1004413 (PMC4199764; doi:10.1371/journal.ppat.1004413)

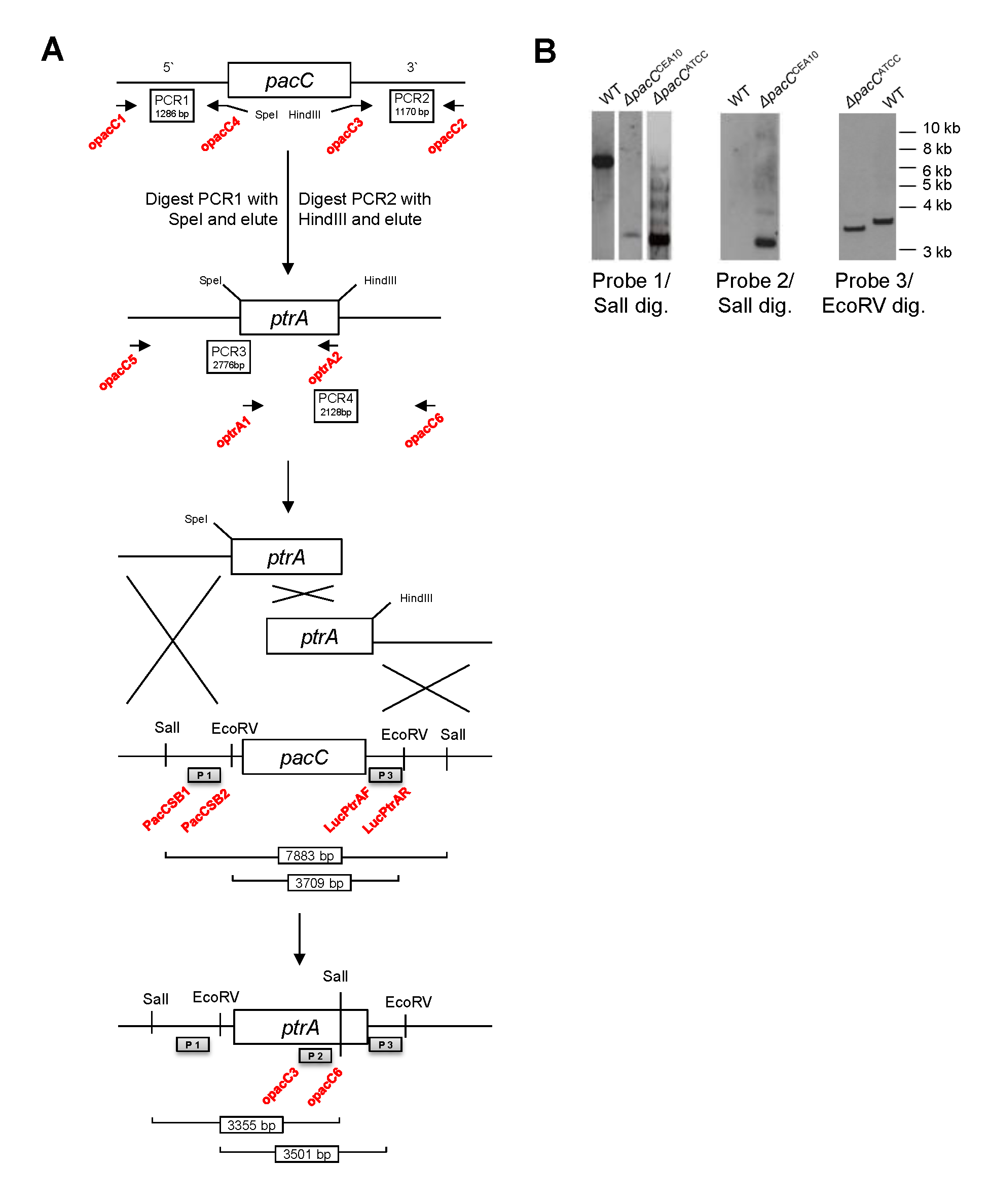

Supplement: Figure S1 — Construction and validation of A. fumigatus ΔpacC mutants. Schematic for the construction of A. fumigatus ΔpacC mutants by gene replacement using a split pyrithiamine resistance marker (A) and Southern blot analysis of A. fumigatus wild-type and ΔpacC mutants (B). According to the first strategy employed, genomic DNA on indicated strains was digested with SalI and probed with two hybridisation probes generated using the oligonucleotides PacCSB1 & PacCSB2 (Probe 1) and LucPtrAF & LucPtrAR (Probe 3) (Table S2). The expected signals for Probe 1 were 7883 bp for the wild-type and 3355 bp for the ΔpacC mutants. For Probe 3, no signal was expected for the wild-type, whereas for a single, homologous integration of the deletion cassette the expected signal was 3355 bp. According to the second strategy employed, genomic DNA of indicated strains was digested with EcoRV and probed with a hybridisation probe generated using oligonucleotides opacC3 and opacC6 (Probe 2) (Table S2). The expected signals for Probe 3 were 3709 bp for the wild-type and 3501 bp for the ΔpacC mutants. (TIFF) [file ppat.1004413.s004.tiff]

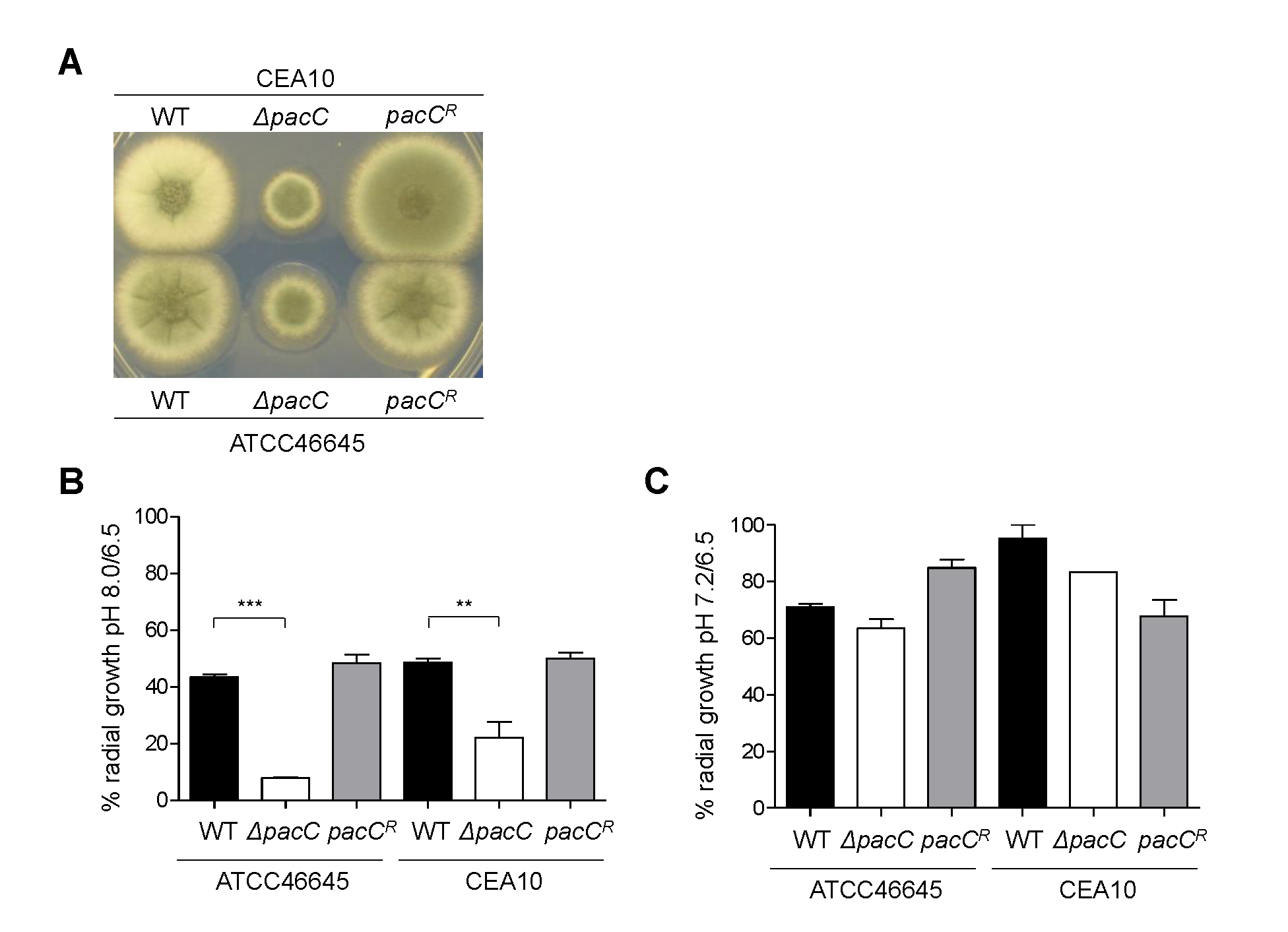

Supplement: Figure S2 — Colonial growth of wild type and ΔpacC mutants. (A) Colonial growth phenotypes on ACM, 48 hr of growth, 103 conidia. (B) Percent radial growth at pH 8.0 relative to pH 6.5, 72 hr of growth, 103 conidia, n = 3, unpaired t test, *** p<0.001 and ** 0.001 <p<0.01. (C) Percent radial growth at pH 7.2 relative to pH 6.5, 72 hr of growth, 103 conidia, n = 3. (TIFF) [file ppat.1004413.s005.tiff]

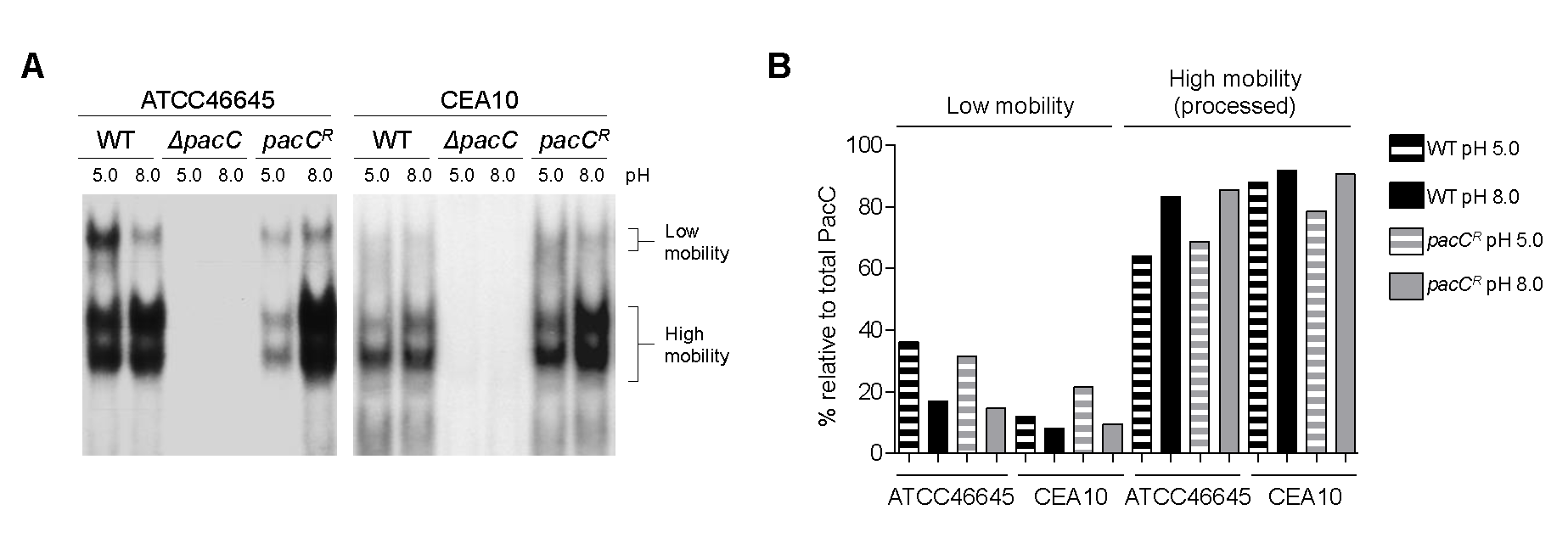

Supplement: Figure S3 — Processing of A. fumigatus PacC. (A) PacC processing after shift to acidic (pH 5.0) and alkaline (pH 8.0) by EMSA. Low mobility and high mobility forms of PacC are indicated on the right side. (B) Densitometry plot of EMSA data expressed, per complex, as a function of total PacC protein. (TIFF) [file ppat.1004413.s006.tiff]

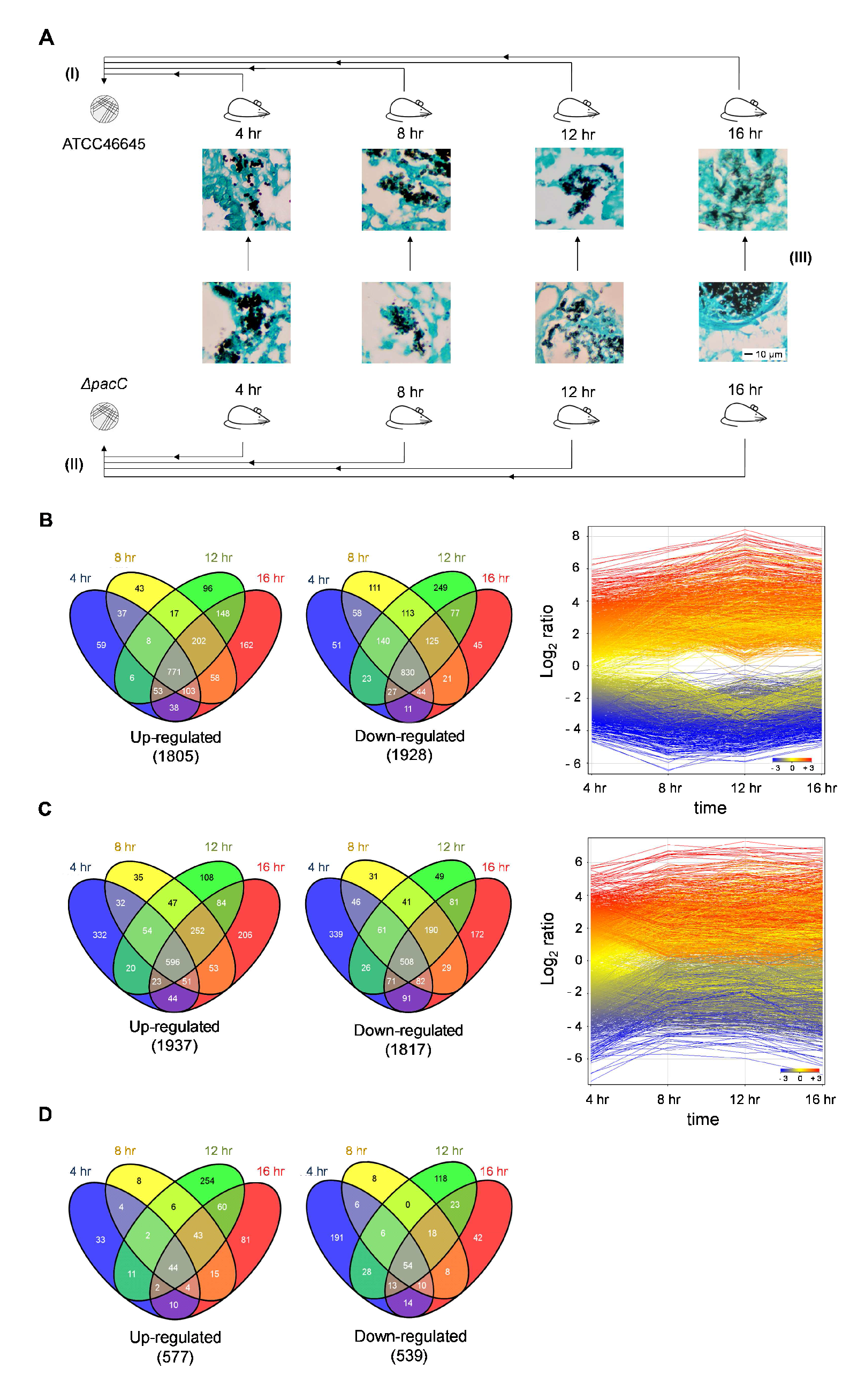

Supplement: Figure S4 — In-host transcriptomic analysis of A. fumigatus wild-type and ΔpacC activities. (A) Experimental design depicting hybridisation schemes. (I) Analysis of wild-type temporal transcription profile during initiation of murine infection comparing gene expression at 4 independent time points (4, 8, 12 and 16 hr) to a common RNA reference extracted from wild-type spores. (II) Analysis of ΔpacC temporal transcription profile during initiation of murine infection comparing gene expression at 4 independent time points (4, 8, 12 and 16 hr) to a common RNA reference extracted from ΔpacC spores. (III) Comparative, stage-specific, analysis of ΔpacC and wild-type transcript profiles during initiation of murine infection (4, 8, 12 and 16 hr). (B, C, D) Up- and down-regulated genes (cut-off ± 1.5) for the wild-type (B), ΔpacC (C) and comparative (D) time courses. Venn diagrams depict the numbers of genes differentially regulated at each time point of the analyses. Profile plots indicate temporal behaviours of all differentially regulated genes relative to the 4 hr time point. (TIFF) [file ppat.1004413.s007.tiff]

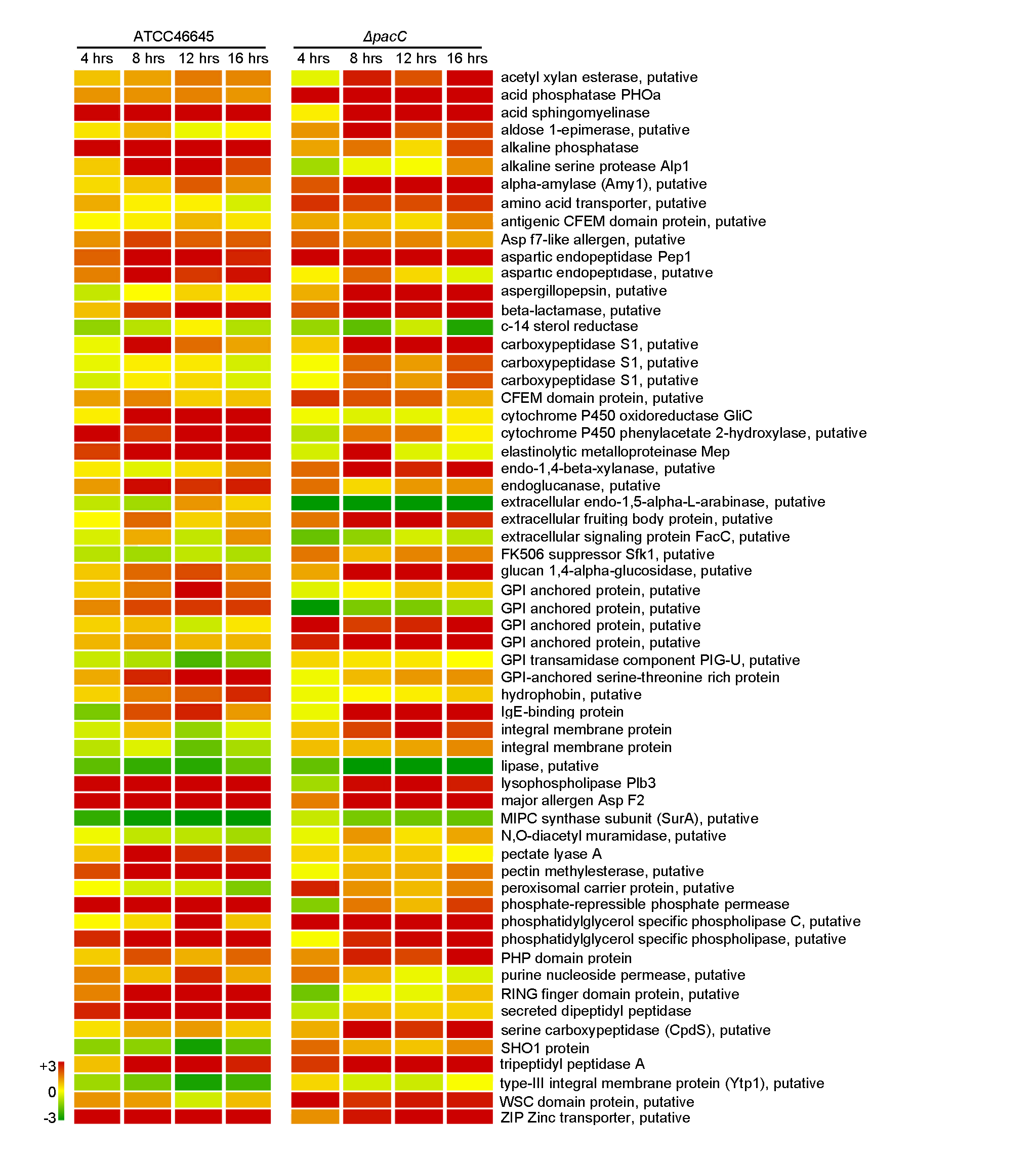

Supplement: Figure S5 — Heat-map depiction of temporal in-host gene expression for wild type and ΔpacC . Heat maps depict results from time-series infection studies and profile gene expression for predicted secreted proteins. Colouration indicates magnitude of log2 ratio relative to RNA reference (ungerminated spores). (TIFF) [file ppat.1004413.s008.tiff]

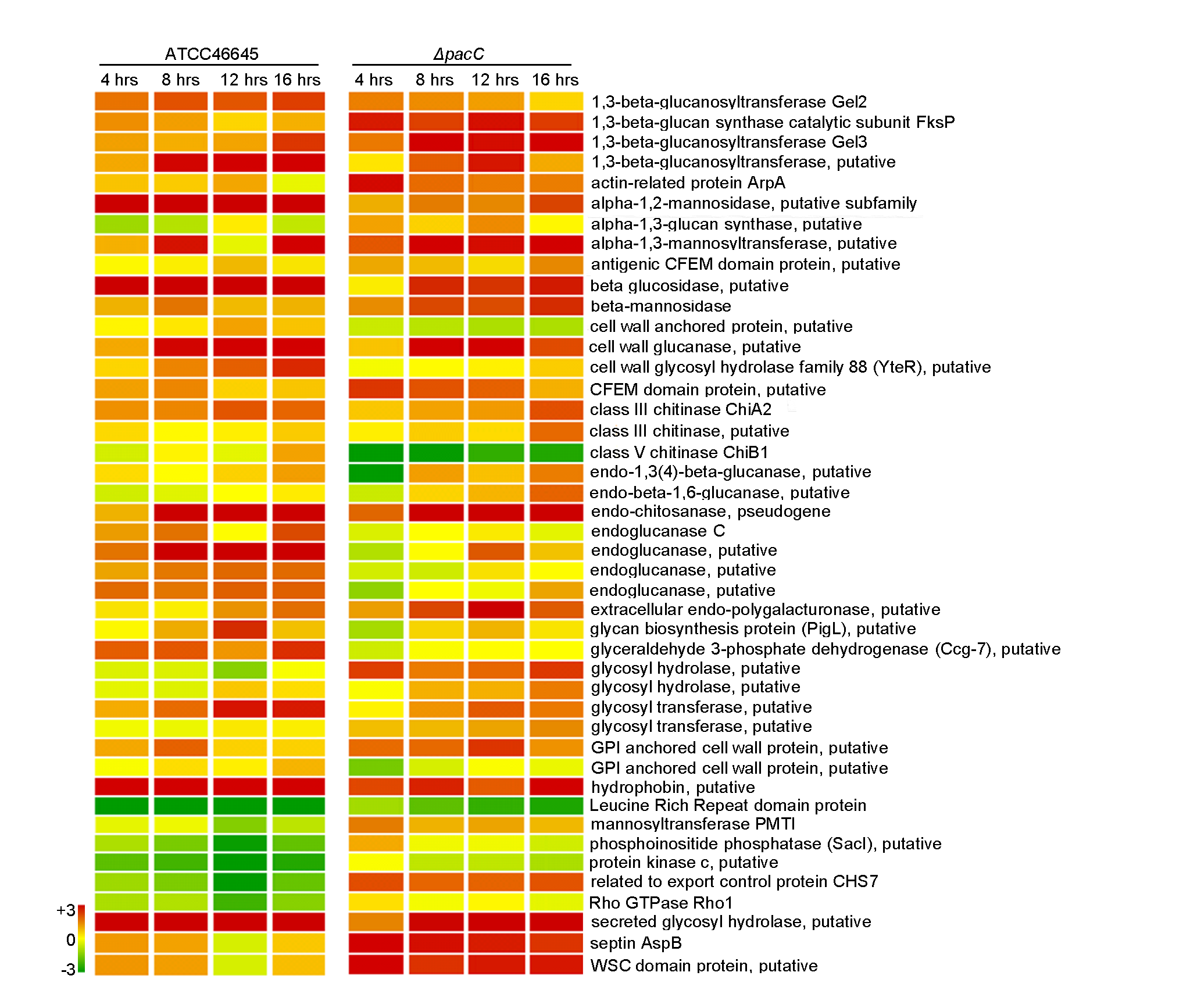

Supplement: Figure S6 — Heat-map depiction of temporal in-host gene expression for wild type and ΔpacC . Heat maps depict results from time-series infection studies and profile gene expression for predicted cell wall biosynthetic gene products. Colouration indicates magnitude of log2 ratio relative to RNA reference (ungerminated spores). (TIF) [file ppat.1004413.s009.tif]

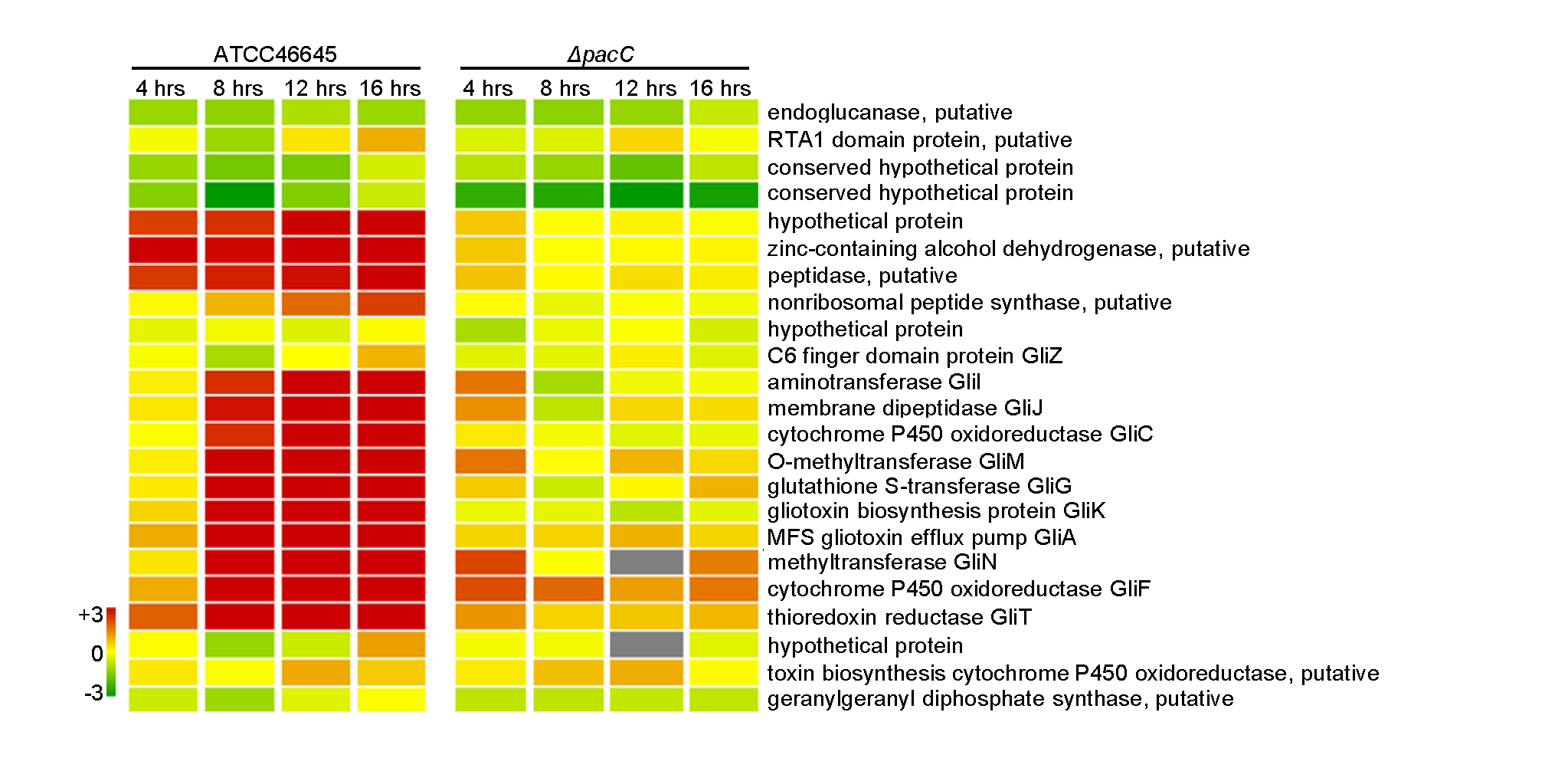

Supplement: Figure S7 — Heat-map depiction of temporal in-host gene expression for wild type and ΔpacC . Heat maps depict results from time-series infection studies and profile gene expression for genes (AFUA_6G09570-AFUA_6G09740; AFUA_6G09745 omitted from the microarray) of the gliotoxin cluster. Colouration indicates magnitude of log2 ratio relative to RNA reference (ungerminated spores). (TIF) [file ppat.1004413.s010.tif]

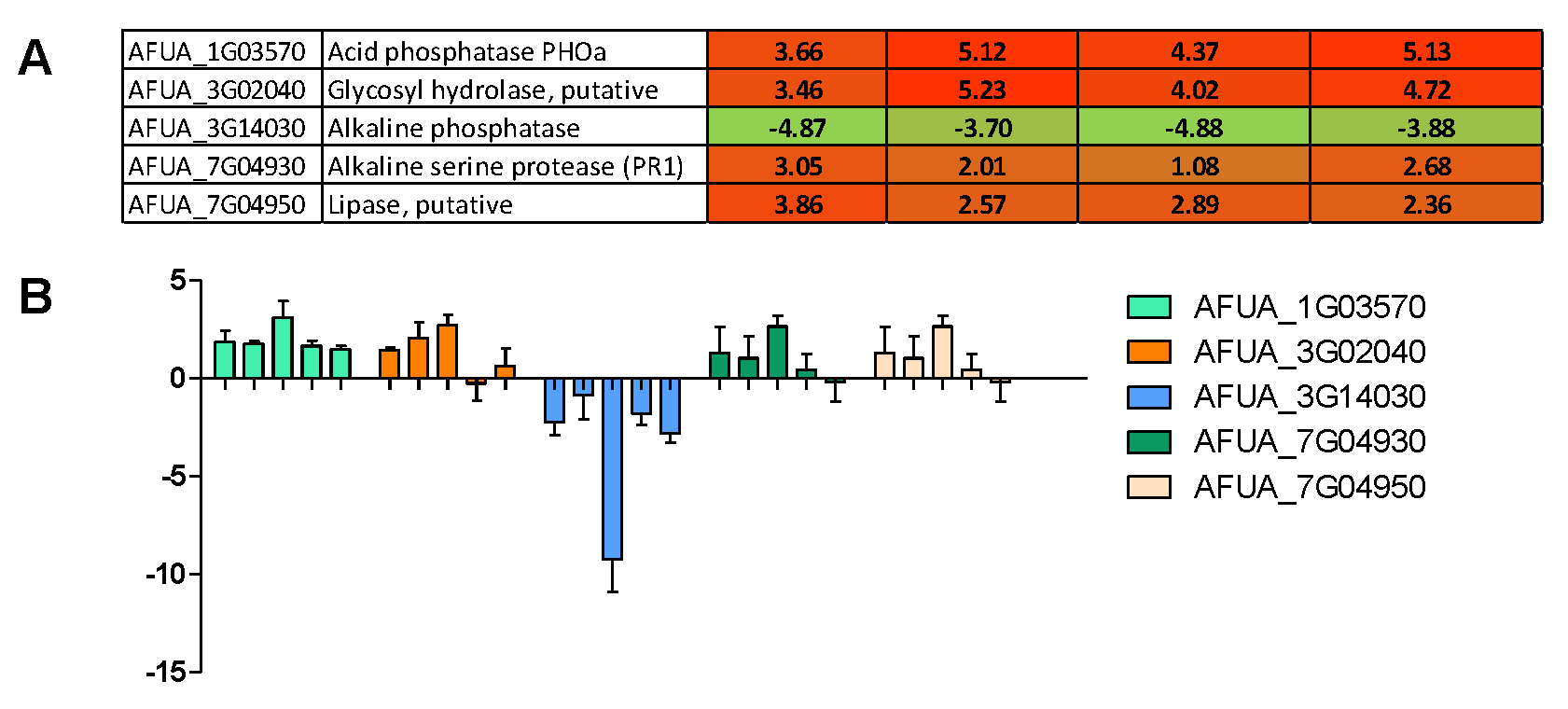

Supplement: Figure S8 — qPCR validation of microarray data. Accuracy of microarray data was independently verified by quantitative RT-PCR on selected transcripts, values (A) and graphical representation (B). Oligonucleotides used for this analysis are detailed in Table S2. Fold change due to treatment (-1/ΔCT) was calculated using A. fumigatus Act1 (AFUA_6G04740) as a house-keeping gene. (TIFF) [file ppat.1004413.s011.tiff]

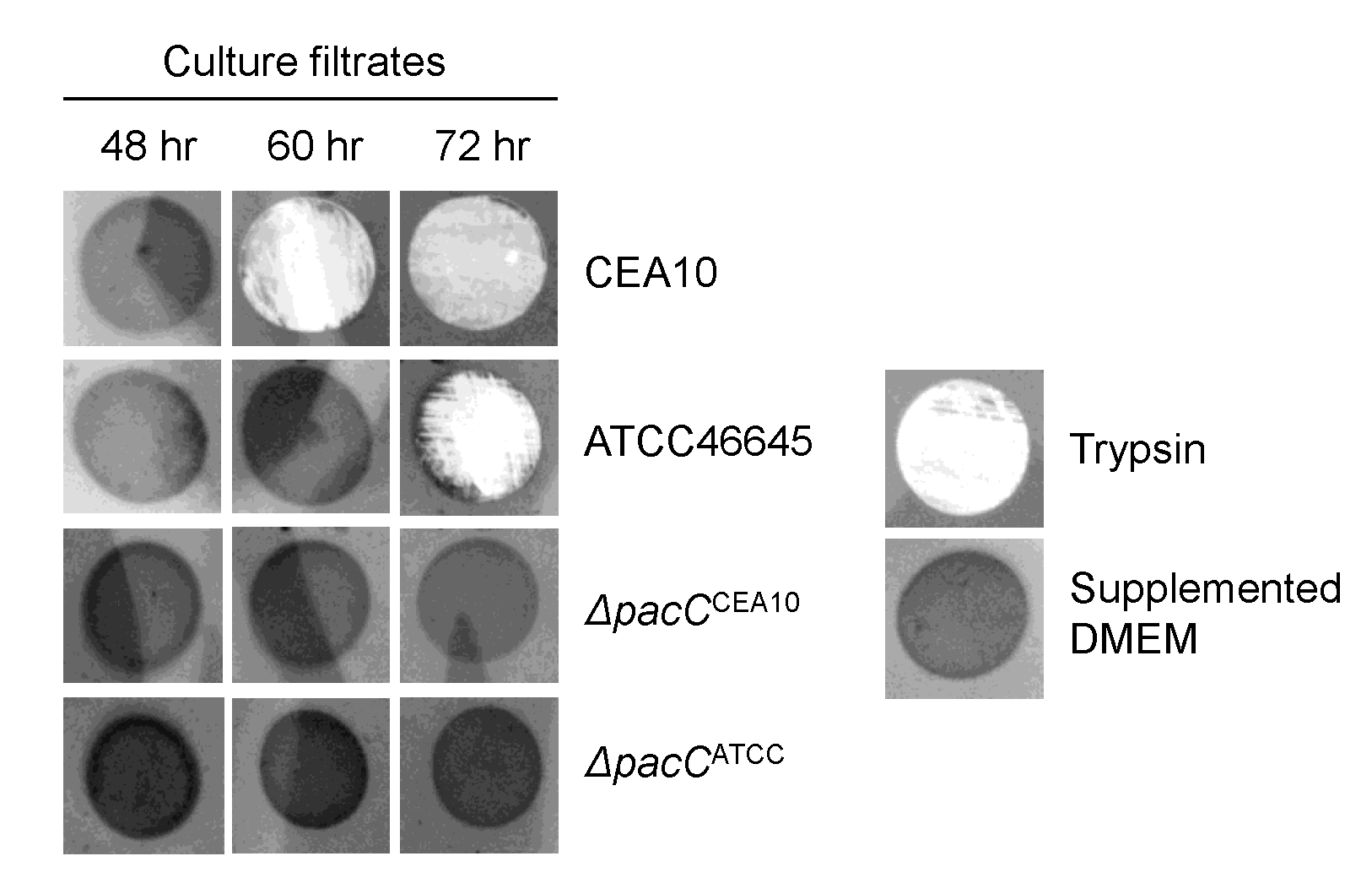

Supplement: Figure S9 — Qualitative analysis of A. fumigatus protease activity in cell culture filtrates. Culture supernatant (50 µl of supplemented DMEM + A549 cell culture, 106 spores/ml) was spotted onto X-ray film, incubated at 42°C and washed after overnight digestion with water. Positive control is represented by trypsin (50 BAEE units). Trypsin hydrolyzes N-benzoyl-L-arginine ethyl ester (BAEE) and one BAEE unit equals the amount of the enzyme determining an increase in absorbance of 0.001 per minute at 25°C and 253 nm. (TIFF) [file ppat.1004413.s012.tiff]

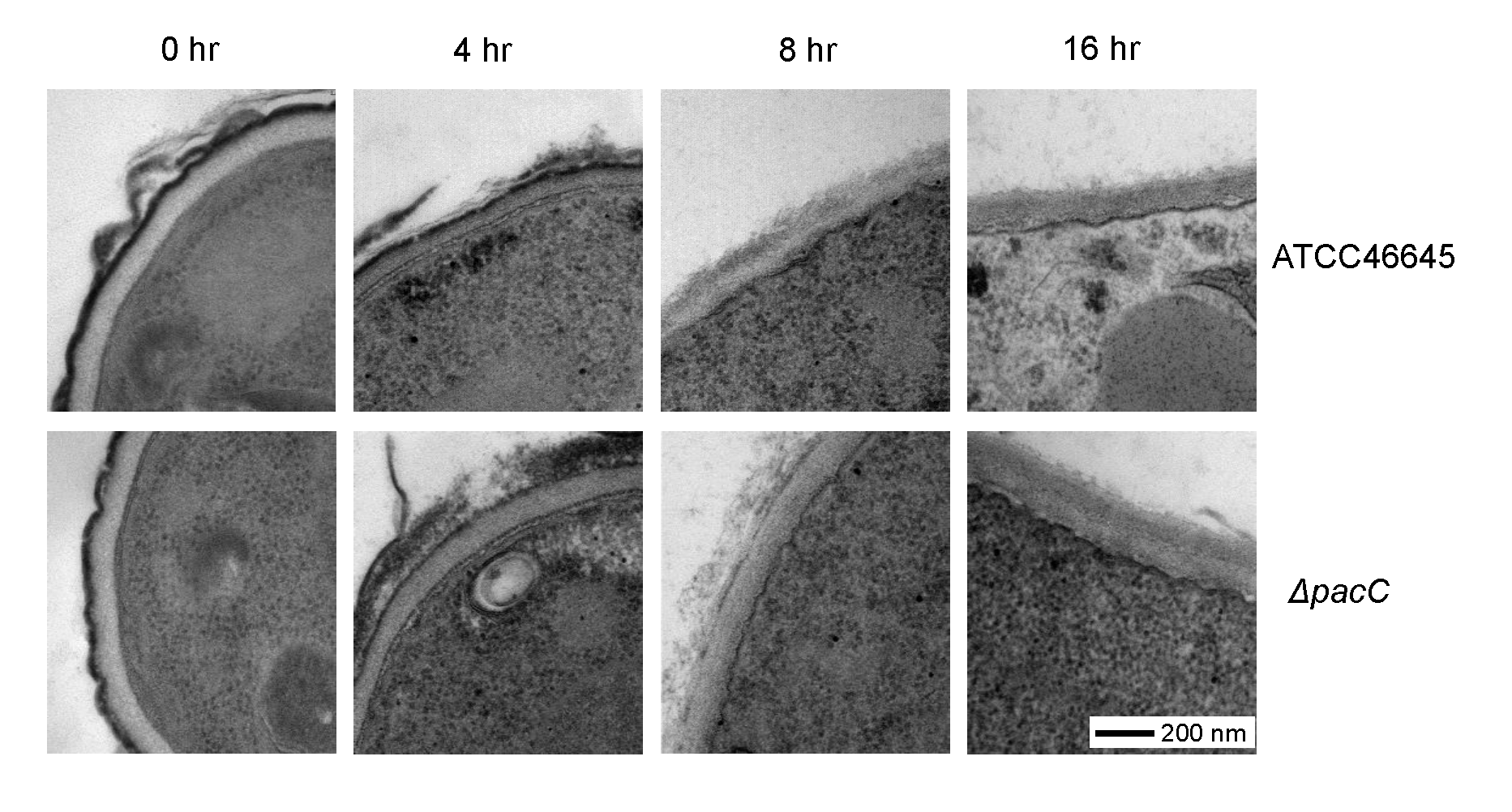

Supplement: Figure S10 — Electron microscopy of A. fumigatus ΔpacC ATCC mutant and the respective parental isolate at 0, 4, 8 and 16 hr of growth. (TIF) [file ppat.1004413.s013.tif]

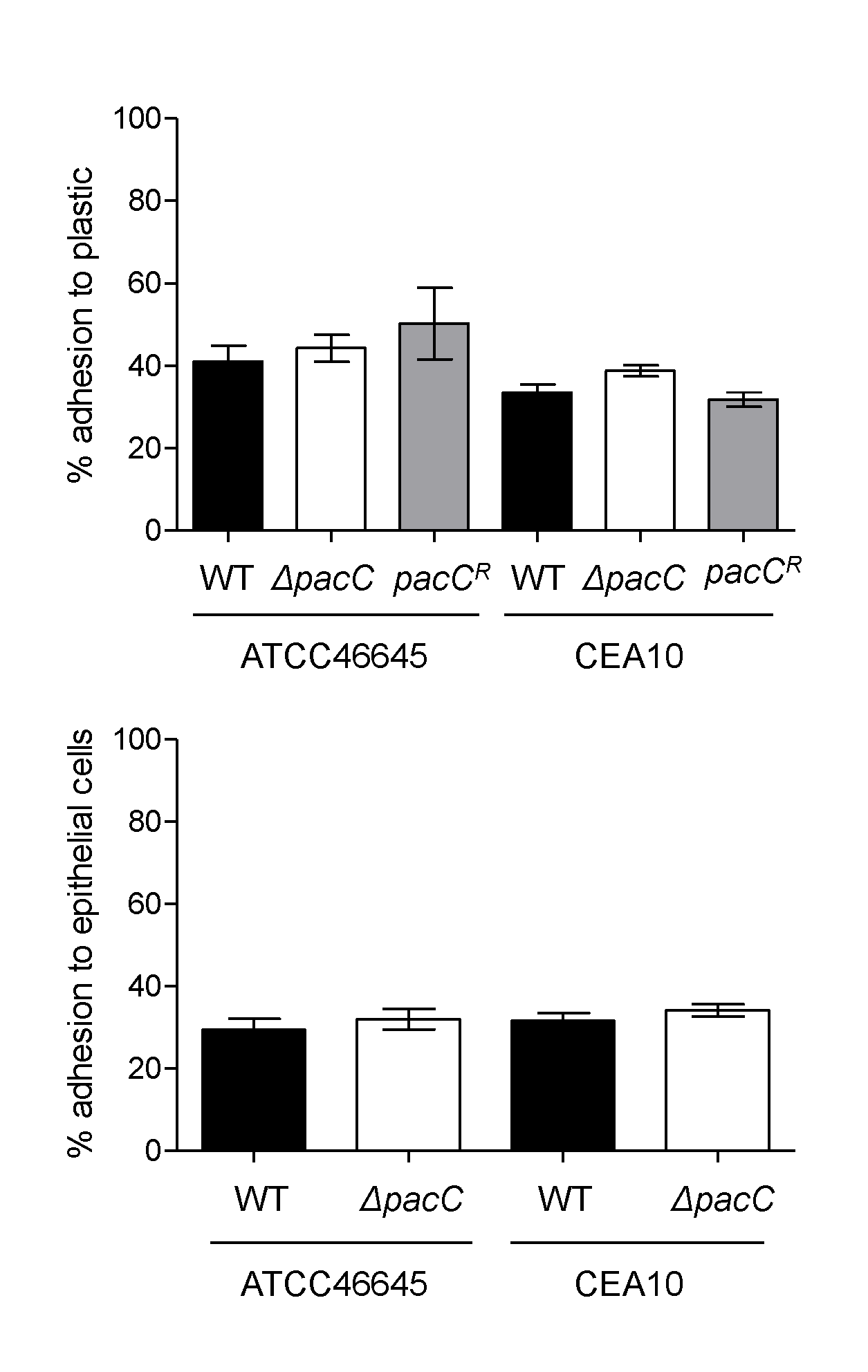

Supplement: Figure S12 — Adhesion of wild type and ΔpacC mutant spores to plastic and A549 monolayers. (TIFF) [file ppat.1004413.s015.tiff]

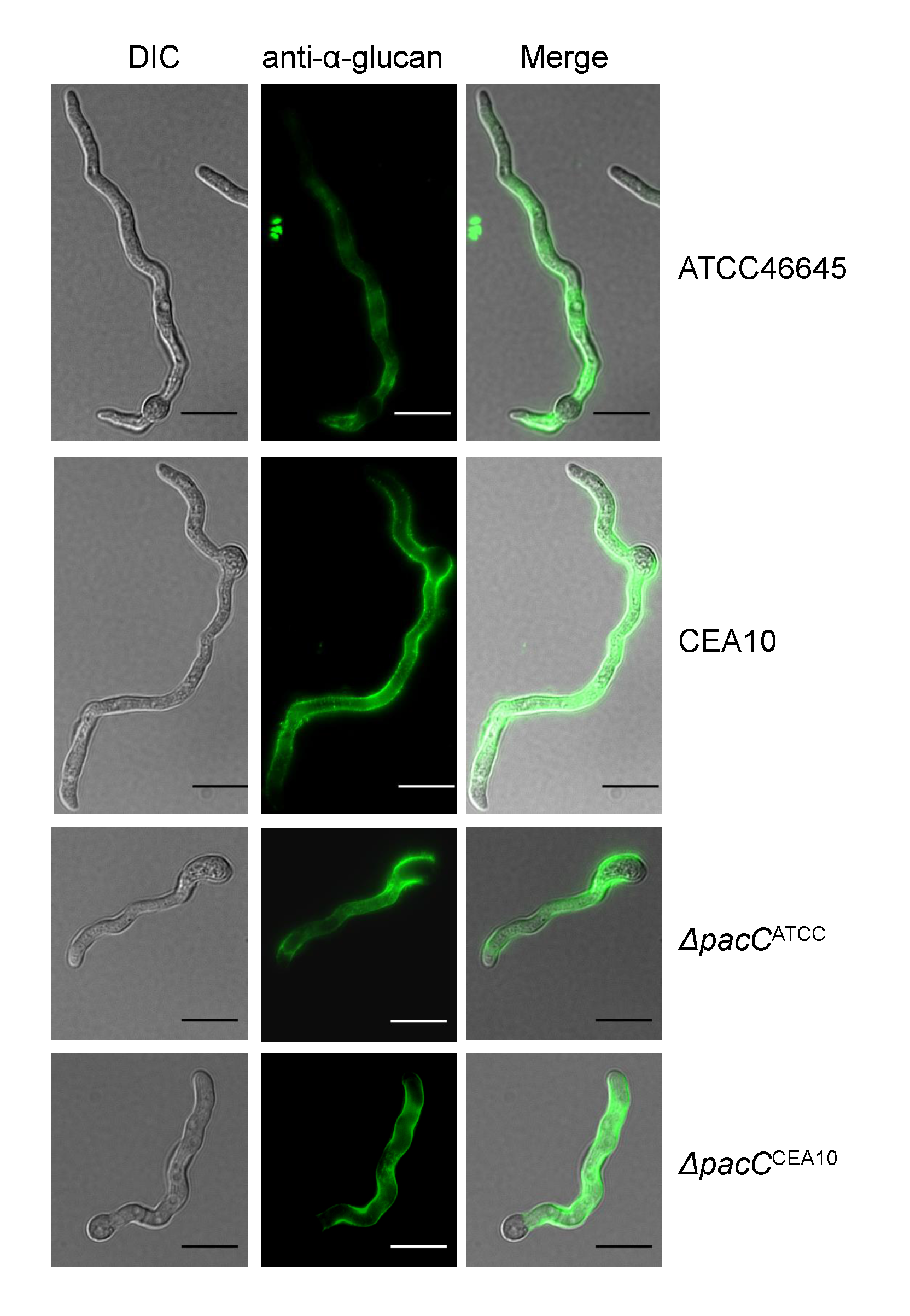

Supplement: Figure S13 — Immunofluorescence-mediated imaging of A. fumigatus α-glucan. (TIF) [file ppat.1004413.s016.tif]
