# Supplementary material for: The pH-Responsive PacC Transcription Factor of Aspergillus fumigatus Governs Epithelial Entry and Tissue Invasion during Pulmonary Aspergillosis
Source: PLoS Pathog. 2014 Oct 16;10(10):e1004413. doi: 10.1371/journal.ppat.1004413 (PMC4199764; doi:10.1371/journal.ppat.1004413)
Supplement: Table S1 — A. fumigatus strains used in this study. (DOCX) [file ppat.1004413.s017.docx]

**Table S1: *A. fumigatus* strains used in this study.**

| **Strain** | **Genotype** | **Source** |
| --- | --- | --- |
| ATCC46645 | Wild-type | ([Hearn and Mackenzie, 1980](#_ENREF_26)) |
| CEA10 | Wild-type | ([Monod et al., 1993](#_ENREF_41)) |
| *ΔpacC*^ATCC^ | ATCC46645; *pacC::ptrA* | Present study |
| *ΔpacC*^CEA10^ | CEA10; *pacC::ptrA* | Present study |
| *pacC*^ATCC(R)^ | *ΔpacC* ATCC46645; *pacC* | Present study |
| *pacC*^CEA10(R)^ | *ΔpacC* CEA10; *pacC* | Present study |
