# Supplementary material for: The pH-Responsive PacC Transcription Factor of Aspergillus fumigatus Governs Epithelial Entry and Tissue Invasion during Pulmonary Aspergillosis
Source: PLoS Pathog. 2014 Oct 16;10(10):e1004413. doi: 10.1371/journal.ppat.1004413 (PMC4199764; doi:10.1371/journal.ppat.1004413)
Supplement: Table S2 — Oligonucleotides used in this study. (DOCX) [file ppat.1004413.s018.docx]

**Table S2: Oligonucleotides used in this study.**

| **Gene** | **Primer** | **Sequence 5’ – 3’** |
| --- | --- | --- |
| *pacC* (AFUA_3G11970) | opacC1 | TCTGTGGTGCAACTGGGAGAGG |
| *pacC* (AFUA_3G11970) | opacC2 | AGCACGACGAAGGAATGGAAGG |
| *pacC* (AFUA_3G11970) | opacC3 | AAT*AAGCTT*ACTGGGCTAGTTCGTCTC |
| *pacC* (AFUA_3G11970) | opacC4 | TTC*ACTAGT*CTTTTCTTGGGAGCAGCG |
| *pacC* (AFUA_3G11970) | opacC5 | CTTGGTCTTTCCTGTCGG |
| *pacC* (AFUA_3G11970) | opacC6 | CTGTAGGAAGGGTTGACG |
| *pacC* (AFUA_3G11970) | pacCF | GGTGGCTACCCCACTGCATC |
| *pacC* (AFUA_3G11970) | pacCR | TGCCGTCGTGAGAAGTGTCC |
| *ptrA* (AO090003000090) | optrA1 | GAGGACCTGGACAAGTAC |
| *ptrA* (AO090003000090) | optrA2 | CATCGTGACCAGTGGTAC |
| *pacC* (AFUA_3G11970) | PacCSB1 | cttggtctttcctgtcgg |
| *pacC* (AFUA_3G11970) | PacCSB2 | cttttcttgggagcagcg |
| *ptrA* (AO090003000090) | PtrAF | cttcctgttgatggaatgg |
| *ptrA* (AO090003000090) | PtrAR | gacggcgcatgaccatag |
| *ptrA* (AO090003000090) | PtrAF | TTCCTGCGGTCTGAGCACTG |
| *ptrA* (AO090003000090) | PtrAR | ACCGCCACCTATTGCACGTT |
| AFUA_1G03570 | AFUA_1G03570_F | CTGCCACCAAAAAGAATGTC |
| AFUA_1G03570 | AFUA_1G03570_R | AGGTTTAGAGCGAGTCCCAA |
| AFUA_3G02040 | AFUA_3G02040_F | ATCACCACCAAACACAAAGG |
| AFUA_3G02040 | AFUA_3G02040_R | ATCCCACCACGAGTCCAATA |
| AFUA_3G14030 | AFUA_3G14030_F | CAACTACAAGGATGCCGTGA |
| AFUA_3G14030 | AFUA_3G14030_R | TCGGCCATGTTGAAGAAGAT |
| AFUA_7G04930 | AFUA_7G04930_F | TGCTGTCTTCTAAGCGATCC |
| AFUA_7G04930 | AFUA_7G04930_R | ATGTAACAGTGCGTTGACGG |
| AFUA_7G04950 | AFUA_7G04950_F | TCCAGTTCCAAGGGTCTTTT |
| AFUA_7G04950 | AFUA_7G04950_R | TTGTTGTCAATGCCCTCGAT |
| AFUA_6G04740 | *OJV607* | GTGCAGATTGTCGCCAG |
| AFUA_6G04740 | *OJV608* | GATTGTCGCCAGGGGAAT |
| AFUA_6G04740 | *OJV609* | AAGCGTTTCATTGTCCAGATTA |

Oligonucleotides were purchased from Sigma-Genosys and designed to contain, where necessary, an appropriate restriction site for cloning which are reported in underlined italic.
