# Supplementary material for: The pH-Responsive PacC Transcription Factor of Aspergillus fumigatus Governs Epithelial Entry and Tissue Invasion during Pulmonary Aspergillosis
Source: PLoS Pathog. 2014 Oct 16;10(10):e1004413. doi: 10.1371/journal.ppat.1004413 (PMC4199764; doi:10.1371/journal.ppat.1004413)
Supplement: Table S3 — A. fumigatus phenotypic testing. (DOCX) [file ppat.1004413.s019.docx]

**Table S3: *A. fumigatus* phenotypic testing.**

| **Media** | **Test Condition** | **Comments** |
| --- | --- | --- |
| AMM pH 5.0 | Acidic pH | Buffered with 100 mM Glycolic acid, pH 5.0 |
| AMM pH 6.5 | Neutral pH | Buffered with 100 mM MES, pH 6.5 |
| AMM pH 7.2 | Physiological (host) pH | Buffered with 100 mM Tris-HCl, pH 7.2 |
| AMM pH 8.0 | Alkaline pH | Buffered with 100 mM Tris-HCl, pH 8.0 |
