# Supplementary material for: The pH-Responsive PacC Transcription Factor of Aspergillus fumigatus Governs Epithelial Entry and Tissue Invasion during Pulmonary Aspergillosis
Source: PLoS Pathog. 2014 Oct 16;10(10):e1004413. doi: 10.1371/journal.ppat.1004413 (PMC4199764; doi:10.1371/journal.ppat.1004413)
Supplement: Text S1 — Supplementary material and methods. (DOCX) [file ppat.1004413.s020.docx]

**SUPPLEMENTARY MATERIALS & METHODS**

**Fungal strains, media and treatments.** Where relevant, *A. fumigatus* spores were harvested into sterile H_2_O from cultures grown on solid ACM for 5 days and spore suspensions were filtered using Miracloth (Calbiochem). Conidial suspensions were spun for 10 min at 4000 rpm, and washed twice with sterile H_2_O. Conidial enumeration was performed using a Nikon Eclipse 80i microscope and a hemocytometer. Spores were resuspended to the appropriate concentration in sterile H_2_O, sterile saline (Baxter Healthcare), or cell culture media. *A. fumigatus* cell wall extracts were prepared from 1 × 10^7^ spores/ml spores cultured in supplementedDMEM at 37°C, 5% CO_2_ for 18 hr (parental isolates and reconstituted strains) or 36 hr (*ΔpacC* mutants) to compensate for the heightened branching frequency of the *ΔpacC* isolates.

**Generation of *ΔpacC* mutants.** *A. fumigatus* protoplasts were co-transformed with two DNA constructs, each containing an incomplete fragment of a pyrithiamine resistance gene (*ptrA*) fused to 1.2 kb, and 1.0 kb of 5’ and 3’ *pacC* flanking sequences, respectively. These marker fragments shared a 557-bp overlap within the *ptrA* cassette, which served as a potential recombination site during transformation. To propagate *ΔpacC* mutants, AMM was supplemented with 0.5 μg/ml pyrithiamine (Takara). For reconstitution of the *ΔpacC* mutants with a functional *pacC* copy, a 4.7 kb PCR fragment, amplified using primers opacC5 and opacC6 (Table S2), was subcloned into pGEM (Promega). The resulting 7.7 kb *pacC^R^* plasmid was linearised with BclI and used to transform *A. fumigatus ΔpacC* protoplasts. Transformants were screened for growth using pH 8.0-mediated selection. Mutants were screened by Southern analysis (Figure S1 and Table S2). Complementation of the *ΔpacC* mutant strains cured all phenotypic defects *in vitro*, indicating that the *ΔpacC* mutant phenotypes arises as a direct result of loss of PacC function.

**qPCR validation of microarrays.** Accuracy of microarray data was independently verified by quantitative RT-PCR on selected transcripts (Figure S8). Oligonucleotides used for this analysis are detailed in Table S2. Fold change due to treatment (-1/ΔC_T_) was calculated using *A. fumigatus* Act1 (AFUA_6G04740) as a house-keeping gene.

**Analysis of protease activity in *A. fumigatus* culture filtrates.** For determination of proteolytic activities of *A. fumigatus* culture filtrates, a qualitative assay based on clearance of unprocessed X-ray film material was used.

***A. fumigatus* cell wall analysis.**

*Electron microscopy.* Briefly, cells were collected and the pellets were fixed with 2.5% (v/v) glutaraldehyde in 0.1 M sodium phosphate buffer (pH 7.3) for 24 hr at 4°C. Samples were encapsulated in 3% (w/v) low melting point agarose prior to processing to Spurr resin following a 24 h schedule on a Lynx tissue processor (secondary 1% OsO_4_ fixation, 1% Uranyl acetate contrasting, ethanol dehydration and infiltration with acetone/Spurr resin). Additional infiltration was provided under vacuum at 60°C before embedding in TAAB capsules and polymerising at 60°C for 48 hr. Semi-thin (0.5 µm) survey sections were stained with toluidine blue to identify regions with optimal cell densities. Ultrathin sections (60 nm) were prepared using a Diatome diamond knife on a Leica UC6 ultramicrotome, and stained with uranyl acetate and lead citrate for examination with a Philips CM10 transmission microscope (FEI UK Ltd, Cambridge, UK) and imaging with a Gatan Bioscan 792 camera (Gatan UK, Abingdon, UK).

*Preparation of cell wall extracts for challenge of monolayer integrity and composition analysis.* Briefly, *A. fumigatus* hyphae were collected by centrifugation at 3000 *g* for 5 min, washed once with chilled deionized water, resuspended in deionized water, and physically fractured with glass beads in a FastPrep machine (Qbiogene). The disrupted cells were collected and centrifuged at 5000 *g* for 5 min. The pellet, containing the cell debris and walls, was washed five times with 1M NaCl, resuspended in buffer (500 mM Tris-HCl buffer, pH 7.5, 2% [wt/vol] SDS, 0.3 M β-mercaptoethanol, and 1 mM EDTA), boiled at 100°C for 10 min, and freeze-dried. For quantification of glucan, mannan, and chitin, cell walls were acid hydrolyzed with 2 M trifluoroacetic acid at 100°C for 3 hr. The acid was evaporated at 60-65°C, and the samples were washed with deionized water and resuspended again in deionized water. The hydrolyzed samples were analyzed by high-performance anion exchange chromatography with pulsed amperometric detection (HPAEC-PAD) in a carbohydrate analyzer system from Dionex (Surrey, United Kingdom). The total concentration of each cell wall component was expressed as µg per mg of dried cell wall, determined by calibration from the standard curves of glucosamine, glucose, and mannose monomers, and converted to a percentage of the total cell wall.

**Adhesion assay.** Six-well culture plates were seeded to obtain confluent monolayers of A549 epithelial cells. In the absence (adherence to plastic) or in the presence of epithelial cells, plates were infected with 200 spores and incubated for 30 minutes. Following incubation, wells were washed 3 times with 2 mL of PBS and overlaid with solid ACM agar for culturing and enumeration. Experiments were performed in biological and technical triplicates.

**Nystatin susceptibility.** Susceptibility of the strains to nystatin was verified by performing the nystatin protection assay in the absence of epithelial cells. Serial dilutions of samples were plated in ACM plates to verify total killing.
